# Sub-5 nm single crystalline organic p–n heterojunctions

**DOI:** 10.1038/s41467-021-23066-3

**Published:** 2021-05-13

**Authors:** Mingchao Xiao, Jie Liu, Chuan Liu, Guangchao Han, Yanjun Shi, Chunlei Li, Xi Zhang, Yuanyuan Hu, Zitong Liu, Xike Gao, Zhengxu Cai, Ji Liu, Yuanping Yi, Shuai Wang, Dong Wang, Wenping Hu, Yunqi Liu, Henning Sirringhaus, Lang Jiang

**Affiliations:** 1 Beijing National Laboratory for Molecular Sciences, Institute of Chemistry Chinese Academy of Sciences, Beijing, China; 2 Key laboratory of Material Chemistry for Energy Conversion and Storage, Ministry of Education, School of Chemistry and Chemical Engineering, Huazhong University of Science and Technology, Wuhan, China; 3 State Key Laboratory of Optoelectronic Materials and Technologies and the Guangdong Province Key Laboratory of Display Material and Technology, School of Electronics and Information Technology, Sun Yat-sen University, Guangzhou, China; 4 Key Laboratory for Micro-Nano Optoelectronic Devices of Ministry of Education, School of Physics and Electronics, Hunan University, Changsha, China; 5 Shanghai Institute of Organic Chemistry, Chinese Academy of Sciences, Shanghai, China; 6 Beijing Key Laboratory of Construction Tailorable Advanced Functional Materials and Green Applications, School of Materials Science & Engineering, Beijing Institute of Technology, Beijing, China; 7 Department of Mechanical and Energy Engineering, Southern University of Science and Technology, Shenzhen, China; 8 College of Science, Tianjin University, Tianjin, China; 9 Cavendish Laboratory, University of Cambridge, Cambridge, UK; 10 University of the Chinese Academy of Sciences, Beijing, China

**Keywords:** Electronic devices, Electronic properties and materials

## Abstract

The cornerstones of emerging high-performance organic photovoltaic devices are bulk heterojunctions, which usually contain both structure disorders and bicontinuous interpenetrating grain boundaries with interfacial defects. This feature complicates fundamental understanding of their working mechanism. Highly-ordered crystalline organic p–n heterojunctions with well-defined interface and tailored layer thickness, are highly desirable to understand the nature of organic heterojunctions. However, direct growth of such a crystalline organic p–n heterojunction remains a huge challenge. In this work, we report a design rationale to fabricate monolayer molecular crystals based p–n heterojunctions. In an organic field-effect transistor configuration, we achieved a well-balanced ambipolar charge transport, comparable to single component monolayer molecular crystals devices, demonstrating the high-quality interface in the heterojunctions. In an organic solar cell device based on the p–n junction, we show the device exhibits gate-tunable open-circuit voltage up to 1.04 V, a record-high value in organic single crystalline photovoltaics.

## Abstract (web summary)

Realizing organic p–n junctions based on ordered crystalline materials with dimensions comparable to the exciton diffusion length of most organic semiconductors remains a challenge. Here, the authors report a strategy to form molecular monolayer crystal-based p–n junctions with thickness below 5 nm.

## Introduction

Semiconductor p–n heterojunctions are essential building blocks for various optoelectronic devices and important platforms for investigation of device physics^1–3^, though most of the p–n heterojunctions by far are based on inorganic semiconductors^4–15^. Taking organic photovoltaic (OPV) devices as an example, critical physical processes such as exciton dissociation, which essentially dominate the efficiency of OPVs, occurs at the p–n heterojunction interfaces. However, the fundamental physical mechanisms regarding these processes are still under debate. This is because the exciton diffusion length is typically about 5–20 nm in OPVs^16,17^, while the thickness of p–n junction is typically higher than this length. Therefore, semiconductor layers outside the exciton diffusion range bring challenges for probing the exciton-related processes underneath. On the other hand, when thickness of the p–n junction is downscaled to the molecular level, excitons generated by photon absorption would be present directly at the p–n junction interface with low loss, and then probably completely dissociate into free holes and electrons. It has been experimentally validated higher device performance could be obtained when size of micro-phase domain decreased^18,19^, and the optimized micro-phase domain size might vary from case to case depending on the materials used. However, the bulk heterojunctions inevitably comprise both structure disorders and complex interpenetrating grain boundaries with interfacial defects, which poses difficulties for elucidating the exciton physics in OPV studies. Hence, achieving highly ordered crystalline p–n heterojunctions with atomically well-defined interface at monolayer thickness limit, is a powerful strategy for studying exciton physics without the limitations imposed by exciton diffusion lengths, as well as an efficient way to reveal the fundamental mechanisms in organic optoelectronic devices. Organic p–n junctions consisting of monolayer molecule crystals (MMCs) combine the advantages of MMCs and crystalline heterojunctions^20–36^, which not only have the inherently efficient charge carrier transport in molecular crystals, but also have bilayer thickness with atomically sharp junction interface^15^, providing a perfect solution to the above-mentioned challenges. However, the direct growth of such thin single-crystalline p–n heterojunctions remains a huge challenge, which significantly limits their applications in organic optoelectronic devices.

## Results

### Growth and characterization of MMCs based p–n heterojunctions

**Fig. 1:**
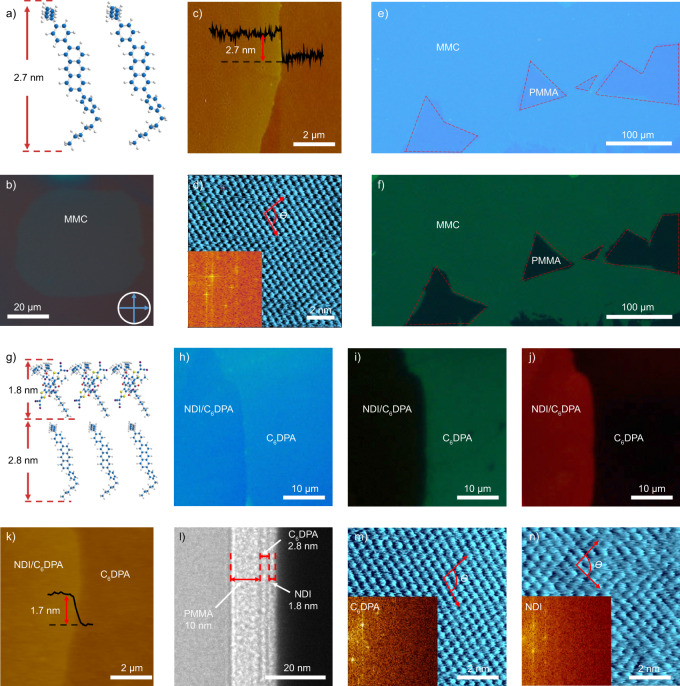
Characterization of 2,6-bis(4-hexylphenyl)anthracene (C_6_DPA) monolayer molecule crystals (MMCs) and bimolecular layer p–n heterojunctions. **a** Molecular arrangement of C_6_DPA MMCs with thickness of around 2.7 nm. Blue represents carbon atom and gray represents hydrogen atom. **b**–**f** Characterizations of C_6_DPA MMCs: **b** cross-polarized optical micrograph; **c** atomic force microscopy (AFM) image; **d** high-resolution AFM (HR-AFM) image (insert: corresponding 2D Fourier transfer pattern); **e**, **f** optical and fluorescent images of large area MMCs, respectively (the dotted line areas refer to poly(methyl methacrylate) (PMMA) and the others C_6_DPA MMCs). **g** Molecular arrangement schematic of bimolecular layer p–n heterojunctions consisting of 2,2′-(2,8-bis(3-hexylundecyl)-1,3,7,9-tetraoxo-1,2,3,7,8,9-hexahydro-[1,3]dithiolo[4′,5′:5,6]benzo[1,2,3,4-lmn][1,3]dithiolo[4,5-f][3,8]phenanthroline-5,11-diylidene)dimalononitrile (NDI) MMC (top, around 1.8 nm thick) and C_6_DPA MMC (bottom, around 2.8 nm thick). Red represents oxygen atom, yellow represents sulfur atom, purple represents nitrogen atom. **h**–**n** Characterizations of NDI-C_6_DPA MMCs based p–n heterojunctions: **h** optical image; **i** fluorescent image at the excitation wavelength of 365 nm (the dark and green regions refer to NDI and C_6_DPA, respectively); **j** fluorescent image at the excitation wavelength of 530 nm (the red and dark regions refer to NDI and C_6_DPA, respectively, the exposure time is elongated for better contrast to show the clear p–n interface); **k** AFM image; **l** cross-sectional transmission electron microscope (TEM) image; **m**, **n** HR-AFM of C_6_DPA MMCs (*b*: 0.48 nm, *c*: 0.48 nm, *θ*: 102.1°) and NDI MMCs (*b*: 0.45 nm, *c*: 0.46 nm, *θ*: 99.1°), respectively. The insert images are corresponding 2D Fourier transfer patterns.

**Fig. 2:**
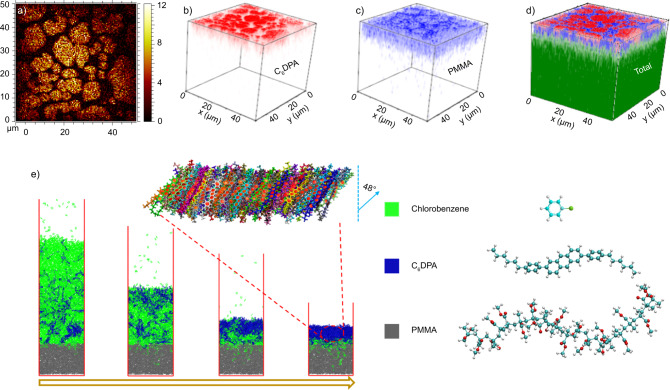
Distribution and self-assembly process of C_6_DPA MMCs. **a**–**d** Time of flight secondary ion mass spectrometry (TOF-SIMS) images of C_6_DPA MMCs: **a** two-dimensional TOF-SIMS images of C_38_H_41_^+^ corresponding to C_6_DPA (the bright colors represent C_6_DPA). **b**–**d** 3D analysis of C_38_H_41_^+^ (C_6_DPA, red colors), C_5_H_9_O_2_^+^ (PMMA, blue colors), respectively and total ions (the green colors represent silicon), respectively, as a function of depth. **e** Molecular dynamics simulations of the formation of MMCs. The left scheme illustrates the formation process. The zoomed-in image on the top is the C_6_DPA molecular stacking model.

The low yield of MMCs and lack of a general growth strategy significantly hinders the mass production of MMCs and limits their further application in p–n heterojunctions. Thus, to overcome this challenge is urgent. In our current study, we proposed a controllable two-dimensional space phase separation method, and MMCs are obtained from a solution mixture of poly(methyl methacrylate) (PMMA) and 2,6-bis(4-hexylphenyl)anthracene (C_6_DPA, Fig. 1a)^29^. By optimizing the parameters, such as the concentration of PMMA and C_6_DPA weight concentration in the solution system (Supplementary Note 1 and Supplementary Figs. 1–7), large scale MMCs could be obtained when the thickness of the blended films is tuned under 10 nm by adopting 4 mg mL^−1^ PMMA and 20 wt.% of C_6_DPA, and the control of the concentrations is vital for the successful fabrication of MMCs. The films exhibit uniform color and brightness under cross-polarized microscopic observation (Fig. 1b and Supplementary Fig. 1d, e), typical characteristic of single-crystalline films. Further atomic force microscopy (AFM) measurement evidences a thickness of 2.7 ± 0.1 nm (Fig. 1c), smaller than the DFT calculated molecular length of C_6_DPA (3.2 nm), indicating a tilted angle of around 57.6° (Supplementary Note 2), which is observed in other monolayer^37,38^. Moreover, optical and fluorescent microscopic images indicate the uniformity of crystals with uniform color distribution, and the maximal lateral size of the MMCs reaches up to 0.47 mm (Fig. 1e, f). High-resolution AFM (HR-AFM, Fig. 1d) and grazing incidence wide-angle X-ray scattering (GIWAXS, Supplementary Fig. 8) results demonstrate the MMCs have highly ordered structure, where the lattice constants along the *b* and *c* axes are 0.45 nm and 0.47 nm, respectively, with a *θ* ~ 99.5°, while the coherence length is about 13 nm. It indicates that C_6_DPA MMCs are exclusively distributed on the top of PMMA layer rather than on the surface of SiO_2_ substrates or inside the PMMA, according to photo-induced force microscopy (PiFM) and time of flight secondary ion mass spectrometry (TOF-SIMS, Fig. 2, Supplementary Note 3 and Supplementary Figs. 9 and 10).

Successful preparation of a series of MMCs, such as 2,7-dioctyl[1]benzothieno[3,2-b][1]benzothiophene (C_8_BTBT)^33^, 1,4-bis((5′-hexyl-2,2′-bithiophen-5-yl)ethynyl)benzene (HTEB)^39^ and 2,2′-(2,8-bis(3-hexylundecyl)-1,3,7,9-tetraoxo-1,2,3,7,8,9-hexahydro-[1,3]dithiolo[4′,5′:5,6]benzo[1,2,3,4-lmn][1,3]dithiolo[4,5-f][3,8]phenanthroline-5,11-diylidene)dimalononitrile (NDI^40^) (Supplementary Fig. 11), opens a way to fabricate bimolecular layer p–n junctions by the two-dimensional space phase separation method. One possible strategy to achieve ultra-thin p–n heterojunctions is to use a blend of p-, n-type semiconductors and PMMA in the processes. A p–n heterojunction is indeed obtained by the one-step spin-coating method when a NDI (n-type, 0.2 mg mL^−1^)/C_6_DPA (p-type, 1 mg mL^−1^) blend was employed. However, this strategy has not yet been successful in other p-/n-type semiconductor blends. We suspect that the p–n heterojunctions can be formed by our method only when the two crystals share similar lattice parameters (Fig. 1m, n, Supplementary Fig. 12 and Supplementary Table 1). Different from the p–n heterojunctions fabricated by mechanically transferring, the thickness of the heterojunction prepared by our method can be precisely controlled at a bimolecular level (Fig. 1g, h). Optical images, fluorescent images, and AFM images confirm that the heterojunctions have a distinct boundary (Fig. 1h–k), with the monolayer NDI crystal (1.7 nm) located on the top of the heterojunction. The absorption and fluorescent spectra of the MMCs and bilayer p–n junctions are also recorded and illustrated in Supplementary Note 4 and Supplementary Fig. 13. Transmission electron microscope (TEM) and TOF-SIMS characterizations (Fig. 1l and Supplementary Fig. 14) also indicate the bilayer structure of the heterojunction and the bottom C_6_DPA MMC structure with a thickness of 2.8 nm. The embedded bottom C_6_DPA MMC has been verified by thickness effect on intensity of Raman spectrum (Supplementary Fig. 15).

The two-dimensional space phase separation process would be dominated by multiple factors, including surface energies, evaporation rate of the solvent, and spin speed^41–43^. To validate the versatility of our fabrication strategy, a variety of substrates were used, including SiO_2_, Si, quartz, Hf_2_O_5_, and plastics (polyethylene terephthalate (PET)). Oxygen plasma treatment was used to improve the wetting property of substrates (Supplementary Methods), and similar MMCs of C_6_DPA could be obtained from these substrates (Supplementary Figs. 1h and 7). We also investigate the effect of polymer composition on the formation of MMCs by preparing the samples from mixture of C_6_DPA and other polymers, such as poly(3-hexylthiophene) (P3HT, regiorandom) and poly[2,5-bis(3-tetradecylthiophen-2-yl)thieno[3,2-b]thiophene] (PBTTT-C14). We observe that MMCs could be obtained from the C_6_DPA/P3HT, but not from C_6_DPA/PBTTT-C14 (Supplementary Fig. 16). Given the remarkable difference in crystallinity between P3HT and PBTTT-C14, it suggests that crystallinity of the polymer may play a critical role in the phase separation processes. To validate our hypothesis, we used the P3HT possessing different regioregularity, with crystallinity following P3HT (regular, regioregularity ≥ 95%) > P3HT (regioregular, regioregularity ≥ 90%) > P3HT (regiorandom) for solution mixtures (Supplementary Fig. 17). After spin-coating the mixture, C_6_DPA MMCs were obtained on both regiorandom P3HT and regioregular P3HT, with the former one having better morphology uniformity. In comparison, no MMCs could be observed for the regular P3HT/C_6_DPA mixture (Supplementary Fig. 16). Such results imply that amorphous polymers are in favor of forming MMCs.

During the spin-coating process, phase separation between PMMA and C_6_DPA occurred with C_6_DPA located on top of the PMMA films. With the effect of centrifugal force, the formation of thick and multilayer crystals has been suppressed effectively. In order to understand the self-assembly processes of C_6_DPA crystalline films on C_6_DPA/PMMA interface, the dynamics of C_6_DPA molecules with chlorobenzene were imitated by non-equilibrium molecular dynamics simulations, as shown in Fig. 2e^44^. The results show that the C_6_DPA molecules can form a relatively ordered and obliquely oriented monolayer on amorphous PMMA upon solvent evaporation. The formation of C_6_DPA crystalline films began because of the stronger intermolecular interaction between C_6_DPA molecules (π–π) compared with that between C_6_DPA and PMMA (C–H ∙ ∙ ∙ π).

### Device fabrication and performance

**Fig. 3:**
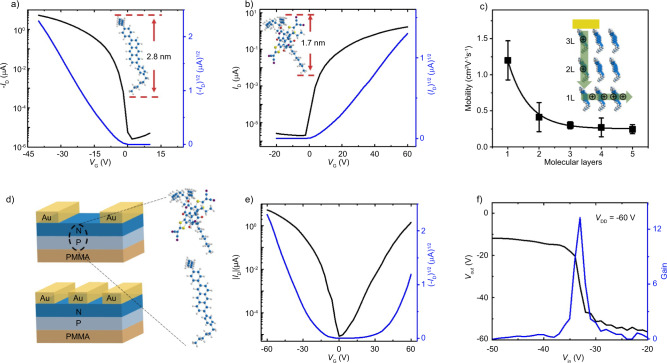
Electrical performance of MMCs and bimolecular layer p–n heterojunction devices. **a**, **b** Transfer curves of p-type C_6_DPA and n-type NDI MMC devices. **c** Mobilities for C_6_DPA devices with different molecular layers (the insert is the working diagram of multilayer crystal device). **d** Schematic diagram of bipolar device and inverter device based on NDI (n type)-C_6_DPA (p type) heterojunctions. **e** Transfer curves of p–n heterojunctions devices. **f** Characteristics of the inverter at *V*_DD_ = −60 V.

The high quality of MMCs promises many potential applications such as organic field-effect transistors (OFETs) and organic circuits. The OFETs based on C_6_DPA MMCs with bottom-gate top-contact device configuration were fabricated. Au stripes were transferred onto MMCs as source and drain electrodes to obtain abrupt metal-semiconductor contacts^29,31,32,34^, and SiO_2_ (300 nm)/PMMA was used as gate dielectric. All electrical characterizations of the C_6_DPA MMC devices were carried out under ambient conditions and all the devices exhibit well-defined transfer characteristics (Fig. 3a and Supplementary Fig. 18). The average mobility of C_6_DPA MMC devices was estimated to be 1.10 cm^2^ V^−1^ s^−1^ with the highest mobility up to 1.61 cm^2^ V^−1^ s^−1^. We have also evaluated the performance of OFETs based on other MMCs (Fig. 3b, Supplementary Fig. 19). For instance, OFETs based on C_8_BTBT MMCs exhibit a maximum mobility of 3.16 cm^2^ V^−1^ s^−1^ (Supplementary Fig. 19b), which is higher than the device with ultrathin active layers obtained from other solution process^45^. The devices with different thickness of C_6_DPA were compared (Fig. 3c, Supplementary Note 5 and Supplementary Fig. 20) by solvent vapor annealing (SVA) of MMCs/PMMA in saturated chlorobenzene atmosphere. Interestingly, the MMC devices possess a higher mobility compared with the thick crystals. The sharp increase in mobilities for C_6_DPA MMCs-based OFETs could be interpreted by the reduced contact resistance^29–32,35^. In addition, OFETs with ultralow operation voltage have also been fabricated on Si substrates by employing the PMMA layer as dielectric, and the mobility was extracted to be about 0.84 cm^2^ V^−1^ s^−1^ at the 2 V operation voltage (Supplementary Fig. 21). Moreover, the ultra-thin structure of MMCs enables the fabrication of ultra-thin, transparent, flexible, and wearable devices (Supplementary Note 6 and Supplementary Figs. 22–24). For the bilayer p–n junction, we also performed electrical measurements on the corresponding OFETs (see Fig. 3d) and successfully observed ambipolar transport behaviors, indicating the good contact between the p-type C_6_DPA and n-type NDI MMCs (see Fig. 3e). The hole and electron mobilities are 0.54 and 0.50 cm^2^ V^−1^ s^−1^ at *V*_DS_ = ± 60 V, respectively, which shows well-balanced ambipolar charge transport performance. With the ambipolar p–n junction transistor device as the basic unit, inverters were also prepared, which exhibit gain of ~14 (see Fig. 3f).

**Fig. 4:**
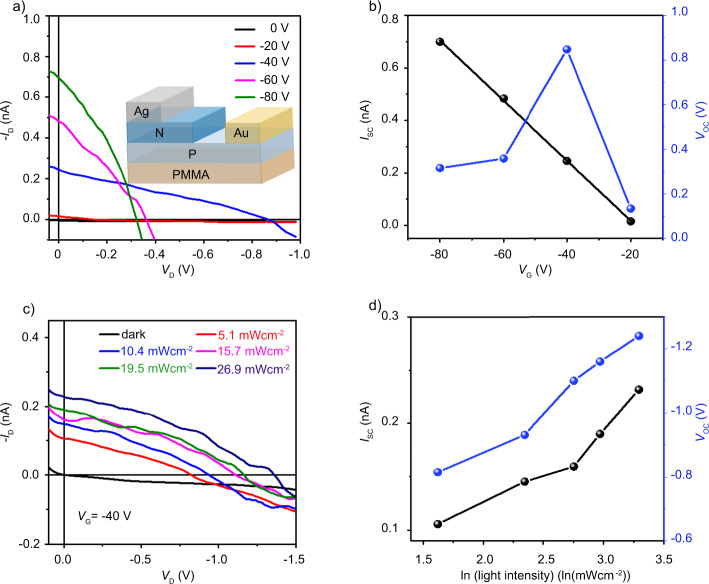
Photovoltaic performance of bimolecular layer NDI (n type)-C_6_DPA (p type) heterojunction devices. **a** Current–voltage characteristics of the device with different gate voltages under the white light illumination (26.9 mW cm^−2^). Insert: the structure diagram of the organic photovoltaic (OPV) device. **c** Current–voltage characteristics of the device with different light intensities at *V*_G_ = −40 V. The dependence of short circuit current (*I*_sc_) and open circuit voltage (*V*_oc_) on gate voltage (**b**) and light intensity (**d**).

The bimolecular layer single-crystalline p–n heterojunctions provide ideal platforms for the understanding and development of OPVs. In order to explore the photovoltaic properties of the NDI/C_6_DPA p–n heterojunction, lateral p–n heterojunction photovoltaic devices were fabricated by transferring Au and Ag films onto the surface of the C_6_DPA and NDI crystals, respectively, to form asymmetric contacts (Fig. 4a). In such a device, when excitons are generated by photon absorption, they would dissociate at the p–n junction interface immediately, following which hole carriers can easily transport along the p-conducting channel to the anode, and simultaneously electrons will move to the upper n-type layer and get collected by the cathode. Figure 4a shows the current–voltage (*I*–*V*) characteristics with different gate bias under the white light illumination (26.9 mW cm^−2^) in ambient conditions. From these data, we extracted the short circuit current (*I*_sc_) and open-circuit voltage (*V*_oc_) versus the gate voltage (*V*_G_) as shown in Fig. 4b. *I*_sc_ increases linearly with the *V*_G_, whereas *V*_oc_ is also tuned by *V*_G_ and it first increases with *V*_G_ and then decreases. Besides, it was seen that *I*_sc_ and *V*_oc_ keep increasing as the light intensity increases from 5.1 to 26.9 mW cm^−2^ (white light, see Fig. 4c and Supplementary Fig. 25), which is consistent with previous studies for normal organic solar cells^46–48^.

The maximum value of *V*_oc_ is 1.04 ± 0.2 V at *V*_G_ = −40 V, which is the highest value achieved in organic single-crystalline p–n heterojunctions so far, to the best of our knowledge. For a reference, the energy level difference between LUMO of n-type and HOMO of p-type is about 1.3 eV (see Supplementary Fig. 26). As *V*_G_ increases, *I*_sc_ increases linearly with the |*V*_G_| and this is attributed to the increased gate-induced hole concentrations and thus the current. The relation between |*V*_oc_| and *V*_G_ is rather complicated. In regular inorganic p–n junctions, the *V*_oc_ increases if the majority carriers in either side of the p–n junction are increased, due to the enlarged difference between the quasi-Fermi levels for electrons and holes. This trend was exactly observed in our device when *V*_G_ was varied from 0 V to −40 V (Supplementary Fig. 27), where the hole concentration gradually increased in the p-channel side. However, at higher gate bias, |*V*_oc_| decreased and this may be explained as follows: the quasi-Fermi level of the p-type C_6_DPA moves closer to HOMO as gate bias increases. However, the gate electric field could also downshift the energy levels of the n-type NDI (including the quasi-Fermi level) and induce carrier tunneling across the junction interface. As a result, the open-circuit voltage *V*_oc_ = (*kT*/*q*)*ln(*I*_ph_/*I*_dark_) decreases. Nevertheless, these results represent interesting physics regarding to the physical processes in OPVs and can be essential to understanding the device physics in OPVs.

## Discussion

In summary, we report a simple and universal method, namely two-dimensional phase separation method, to controllably prepare uniform, high-quality, and large-area MMCs by blending the small molecule semiconductors with amorphous polymers. With this method, C_6_DPA MMCs with a lateral size of more than 400 μm were successfully obtained. More importantly, the method is found to be generally applicable to other small molecular semiconductors such as C_8_BTBT, HTEB, and NDI, and can be performed on various substrates. Furthermore, we report the one-step growth strategy for constructing ultra-thin vertical organic crystalline p–n junctions with atomically clean and sharp interfaces, and demonstrate their application in a prototype photovoltaic device. This study not only provides a simple yet effective solution for the facile fabrication of MMC-based p–n heterojunctions, but also offers a promising strategy to achieve next-generation optoelectronic devices at monolayer limit.

## Methods

### Materials

The C_6_DPA MMCs were prepared by spin-coating the PMMA/C_6_DPA (weight ratio = 4:1) solution on the SiO_2_/Si^++^ substrates at 1500 rpm for 10 s, in which the dielectric layer is composed of SiO_2_ and PMMA (capacitance, 9.5 nFcm^−2^). Other MMCs could also be obtained by this way but from different small molecule/polymer blends, that is 1 mg mL^−1^ HTEB and 4 mg mL^−1^ PMMA for HTEB MMCs, 2 mg mL^−1^ C_8_BTBT and 4 mg mL^−1^ PMMA for C_8_BTBT MMCs and 0.5 mg mL^−1^ NDI and 4 mg mL^−1^ PMMA for NDI MMCs, respectively. The growth processes of the MMCs are described in the Supplementary Methods.

### Measurements

Raman and fluorescent spectra were carried out by WITec alpha300R Confocal Raman Microscope. The microscope images and AFM images of MMCs were carried out by DM4M fluorescent microscope and Nanoscope IIIa instrument (Digital Instruments), respectively. HR-AFM were carried out by Cypher ES Environmental AFM (Oxford Instruments AR). The detailed information of structure characterization of MMCs are provided in the Supplementary Methods.

The electrical characteristics of the devices were measured by Keithley 4200-SCS and Agilent B1500A semiconductor parameter analyzer. The mobility was extracted from the saturated region characteristics by the equation below: $\mu =\frac{2L}{W{{\rm{C}}}_{{\rm{i}}}}\times {\left(\frac{{\rm{d}}\sqrt{{I}_{{\rm{DS}}}}}{{\rm{d}}{V}_{{\rm{G}}}}\right)}^{2}$. As for the bimolecular layer p–n heterojunction devices, the influence of gate bias and light intensity was explored in ambient conditions. The current–voltage (*I*–*V*) characteristics of different gate bias were carried out under the white light illumination (26.9 mW cm^−2^). The influence of light intensity was explored as the light intensity increased from 5.1 to 26.9 mW cm^−2^.

### Simulations

Molecular dynamics (MD) simulations were performed by the Gromacs-4.6.7 software package with the general AMBER force field, which are described in the Supplementary Methods.

### Statistics and reproducibility

MMCs can be obtained with similar results for more than 60 times independent experiment. More than 20 OFETs and 5 inverters and 5 OPVs were prepared, and similar results were obtained.

## Supplementary information

Supplementary information

## Data Availability

The data that support the finding of this study are included within the Article and its Supplementary Information files, or available from the corresponding authors upon reasonable request.
