# Supplementary material for: Sub-5 nm single crystalline organic p–n heterojunctions
Source: Nat Commun. 2021 May 13;12:2774. doi: 10.1038/s41467-021-23066-3 (PMC8119440; doi:10.1038/s41467-021-23066-3)
Supplement: Supplementary file 1 — Supplementary information [file 41467_2021_23066_MOESM1_ESM.pdf]

SUPPLEMENTARY INFORMATION

Sub-5 nm single crystalline organic p-n heterojunctions

Mingchao Xiao[1,2,#], Jie Liu[1,#], Chuan Liu[3], Guangchao Han[1], Yanjun Shi[1], Chunlei Li[1], Xi Zhang[1], Yuanyuan Hu[4], Zitong Liu[1], Xike Gao[5], Zhengxu Cai[6], Ji Liu[7], Yuanping Yi[1]*, Shuai Wang[2]*, Dong Wang[1], Wenping Hu[8], Yunqi Liu[1], Henning Sirringhaus[9] and Lang Jiang[1,10]*

[1]Beijing National Laboratory for Molecular Sciences, Institute of Chemistry Chinese Academy of Sciences, Beijing 100190, China.

[2]Key laboratory of Material Chemistry for Energy Conversion and Storage, Ministry of Education, School of Chemistry and Chemical Engineering, Huazhong University of Science and Technology, Wuhan, 430074, China.

[3]State Key Laboratory of Optoelectronic Materials and Technologies and the Guangdong Province Key Laboratory of Display Material and Technology, School of Electronics and Information Technology, Sun Yat-sen University, Guangzhou 510275, China

[4]Key Laboratory for Micro-Nano Optoelectronic Devices of Ministry of Education, School of Physics and Electronics, Hunan University, Changsha 410082, China.

[5]Shanghai Institute of Organic Chemistry, Chinese Academy of Sciences, Shanghai, 200032, China.

[6]Beijing Key Laboratory of Construction Tailorable Advanced Functional Materials and Green Applications, School of Materials Science & Engineering, Beijing Institute of Technology, 5 South Zhongguancun Street, Beijing, 100081, China.

[7]Department of Mechanical and Energy Engineering, Southern University of Science and Technology, Shenzhen 518055, China.

[8]College of Science, Tianjin University, Tianjin 300072, China.

[9]Cavendish Laboratory, University of Cambridge, JJ Thomson Avenue, Cambridge CB3 0HE, UK.

[10]University of the Chinese Academy of Sciences, Beijing 100049, China.

[#]These authors contributed equally: Mingchao Xiao, Jie Liu.

e-mail: ljiang@iccas.ac.cn, chmsamuel@mail.hust.edu.cn, ypyi@iccas.ac.cn

Supplementary Figures

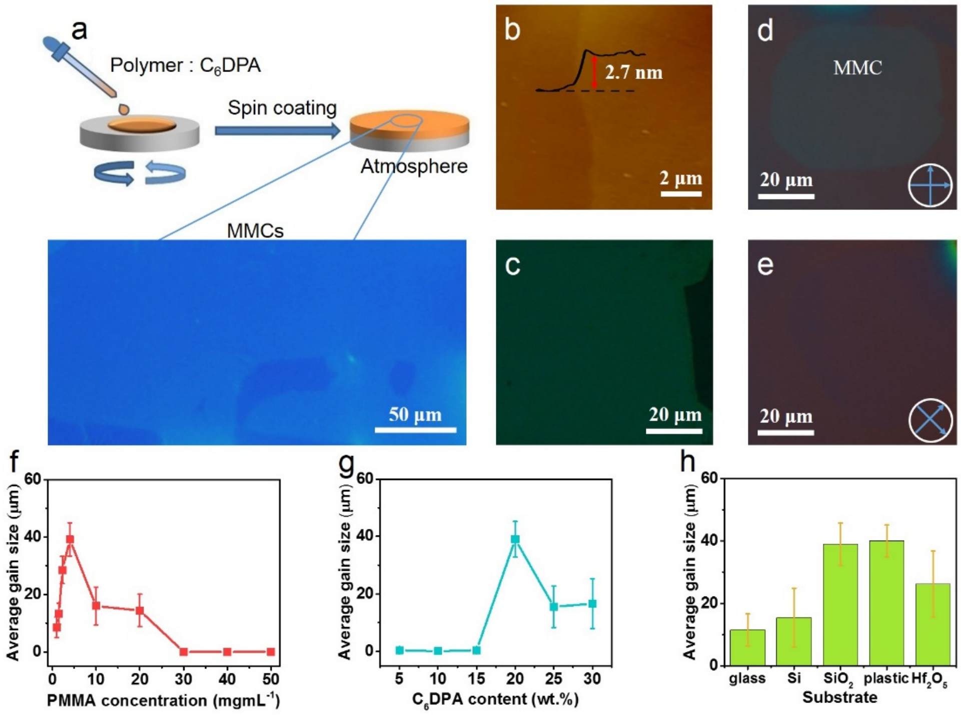

Supplementary **Figure 1. Schematic diagram of the two-dimensional space phase separation method. a**, Schematic illustration of the formation of C_6DPA MMCs (the insert is the optical microscopy image of MMCs). **b**,**c**, AFM and fluorescent images of MMCs, respectively. **d**, **e**, Cross-polarized microscopy images of typical C_6DPA MMC at different sample orientations. **f, g**, Average grain size of C_6DPA MMCs in the film prepared by the mixed solution with (**f**) various PMMA concentration and (**g**) varied C_6DPA content at 4 mgmL^{-1} PMMA; since no MMCs are obtained for high PMMA concentration (>20 mgmL^{-1}) and low C_6DPA concentration (< 20 wt.%), the average grain size is denoted as "0". **h**, Average grain size of C_6DPA MMCs on different substrates. The error bar are the variance of the gain size in 100 µm×100 µm.

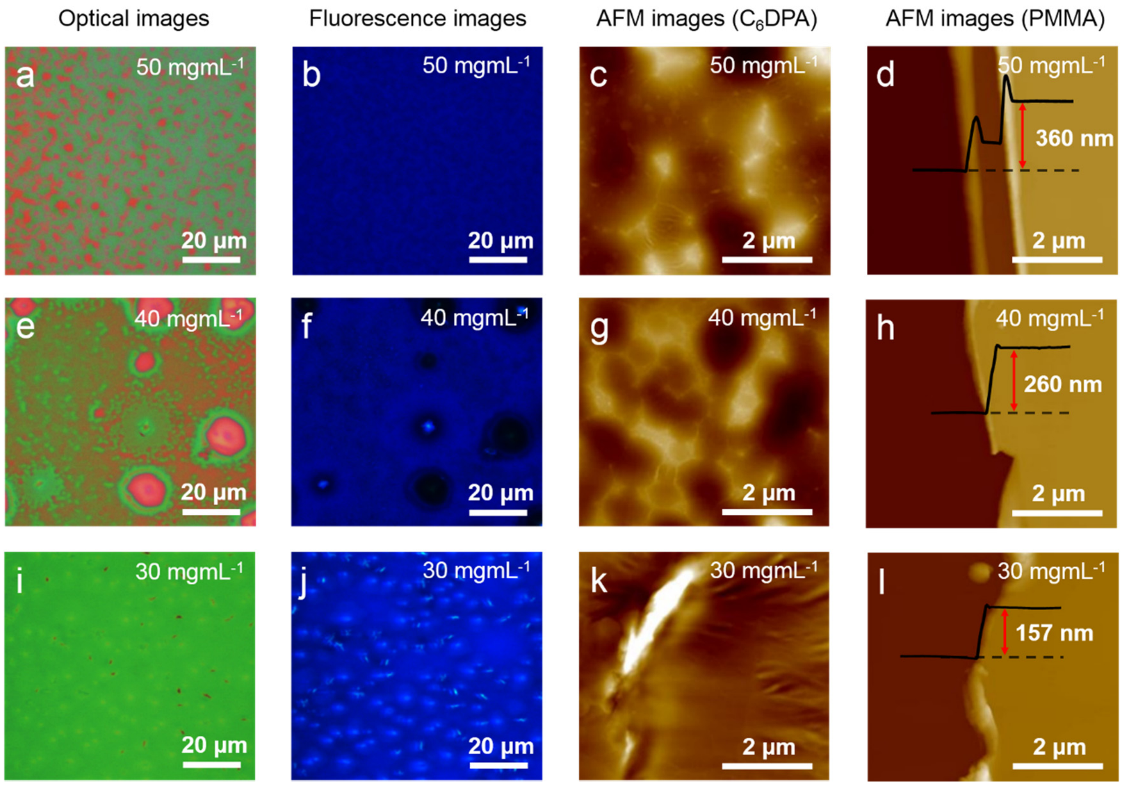

Supplementary Figure 2. Characterization of C_6DPA in marginal zone. The characterization of C_6DPA with a constant concentration of C_6DPA (1 $mgmL^{-1}$) and varied concentration of PMMA as (**a-c**) 50, (**e-g**) 40 and (**i-k**) 30 $mgmL^{-1}$, respectively. The thickness of the mixture film ranges from 157 to 360 nm. The right column (**d, h** and **l**) represents the corresponding AFM images of PMMA in **a**,**e** and **i** respectivley.

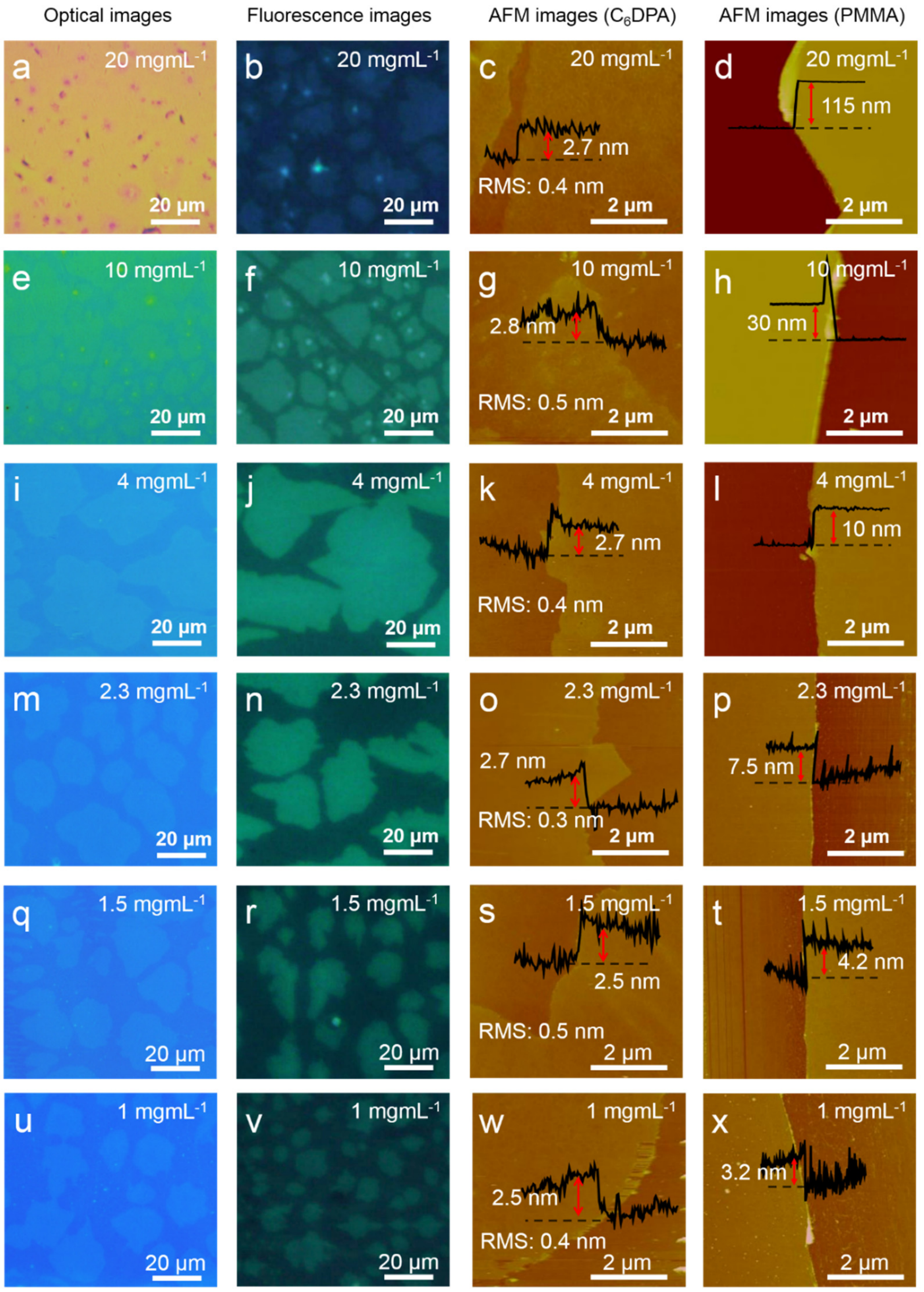

Supplementary Figure 3. Characterization of C_6DPA in marginal zone. The characterization of C_6DPA with a constant concentration of C_6DPA (1 mgmL^{-1}) and varied concentration of PMMA as (**a-c**) 20, (**e-g**) 10, (**i-k**) 4, (**m-o**) 2.3, (**q-s**) 1.5 and (**u-w**) 1 mgmL^{-1}, respectively. The right column (**d, h, l, p, t** and **x**) represents the corresponding AFM images of PMMA in **a**,**e**,**i**,**m**,**q** and **u** respectivley. The thickness of the mixture film decrease from 115 to 3.2 nm.

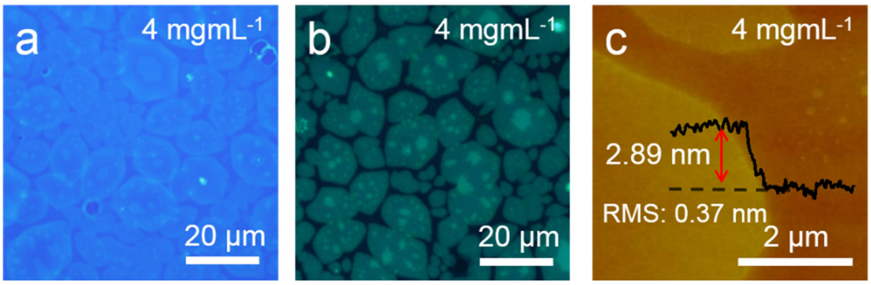

Supplementary Figure 4. Characterization of C_6DPA in central zone. Characterization of C_6DPA MMCs in central zone with the concentration of C_6DPA being 1 mgmL^{-1} and the concentration of PMMA being 4 mgmL^{-1}. **a**, Optical image. **b**, Fluorescent image. **c**, AFM image.

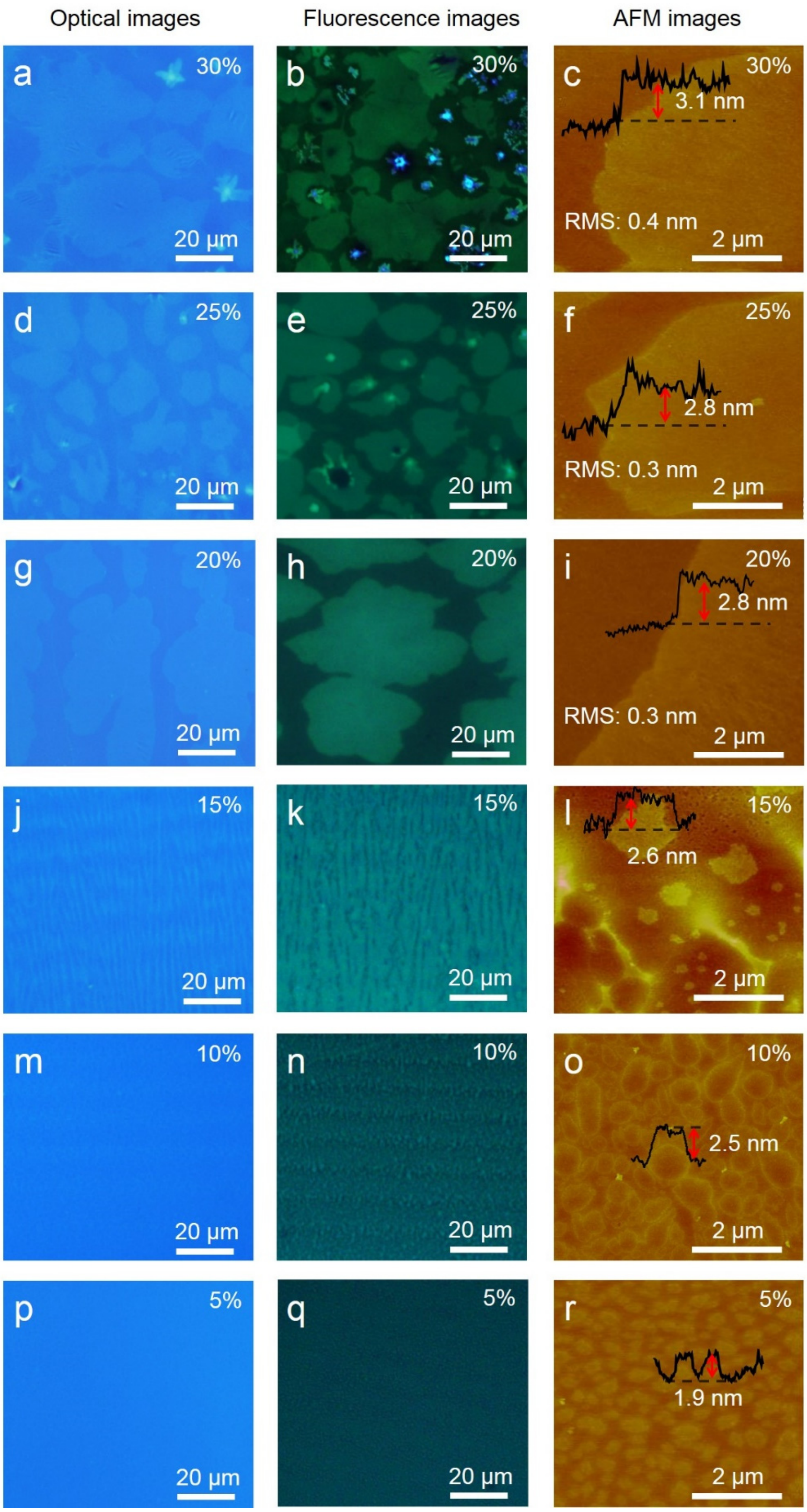

Supplementary **Figure 5. Characterization of C_6DPA MMCs in marginal zone.** The characterization of C_6DPA MMCs in marginal zone with a constant concentration of PMMA (4 mgmL^{-1}) and the varied weight concentration of C_6DPA as (**a-c**) 30 wt.%, (**d-f**) 25 wt.%, (**g-i**) 20 wt.%, (**j-l**) 15 wt.%, (**m-o**) 10 wt.% and (**p-r**) 5 wt.%, respectively.

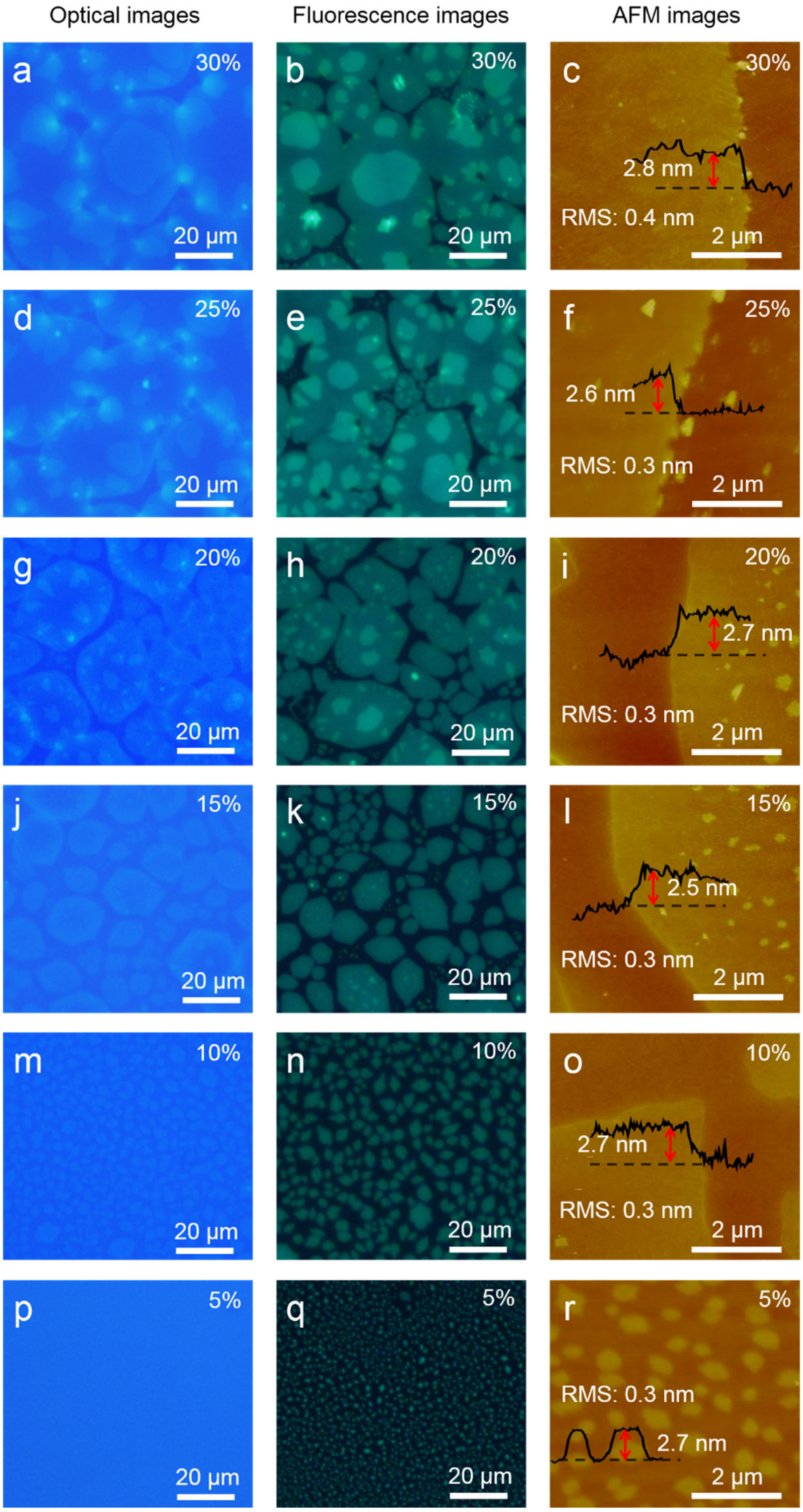

Supplementary Figure 6. Characterization of C_6DPA MMCs in central zone. The characterization of C_6DPA MMCs in central zone with a constant concentration of PMMA (4 mgmL^{-1}) and varied weight concentration of C_6DPA as **(a-c)** 30 wt.%, **(d-f)** 25 wt.%, **(g-i)** 20 wt.%, **(j-l)** 15 wt.%, **(m-o)** 10 wt.% and **(p-r)** 5 wt.%, respectively.

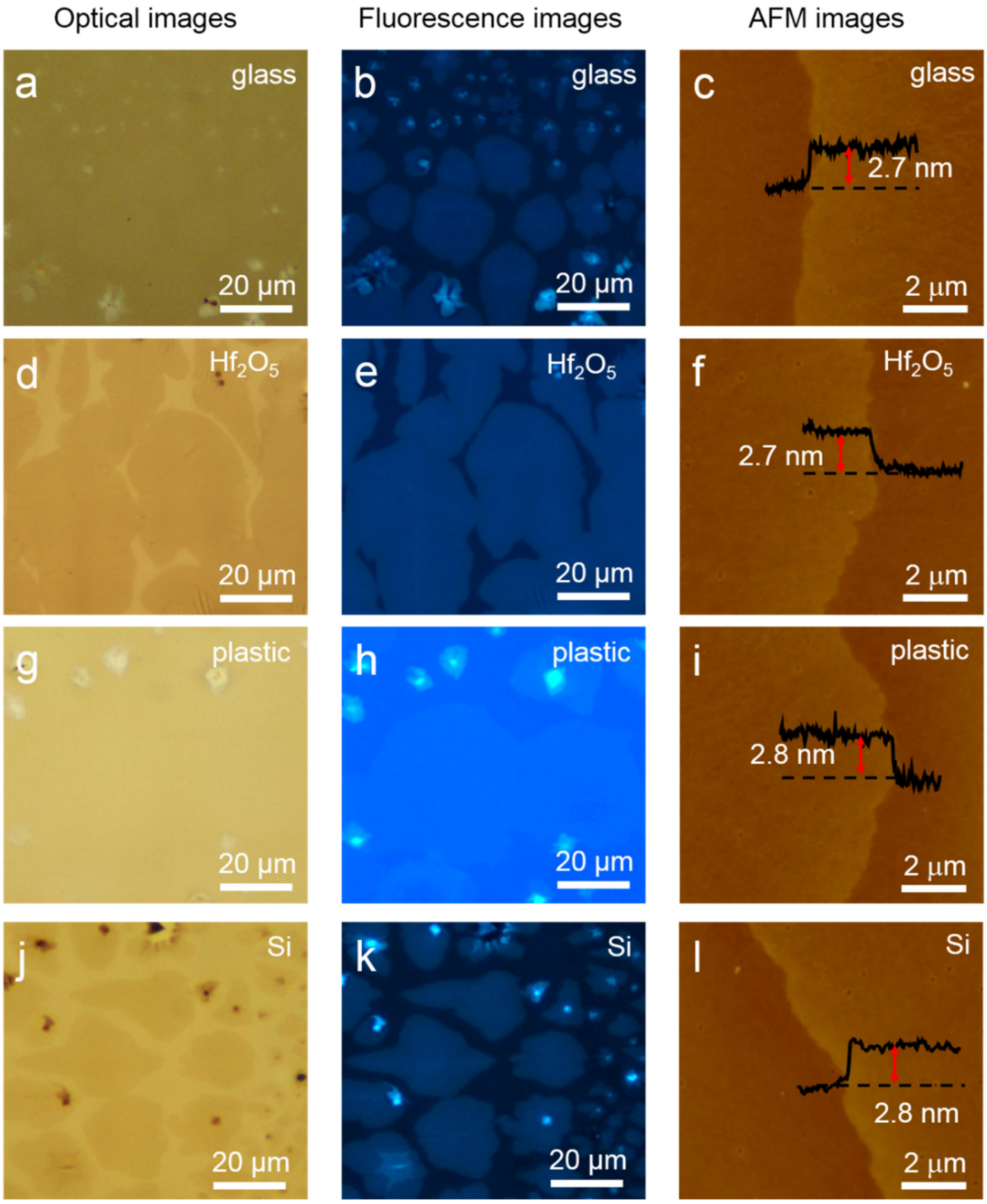

Supplementary Figure 7. The C_6DPA MMCs prepared on different substrates with oxygen plasma treatment. The MMCs on (**a-c**) glass, (**d-f**) Hf_2O_5, (**g-i**) plastic (Polyethylene terephthalate (PET)) and (**j-l**) Si, respectively. The concentration of PMMA is 4 mgmL^{-1} and weight concentration of C_6DPA is 20 wt.%.

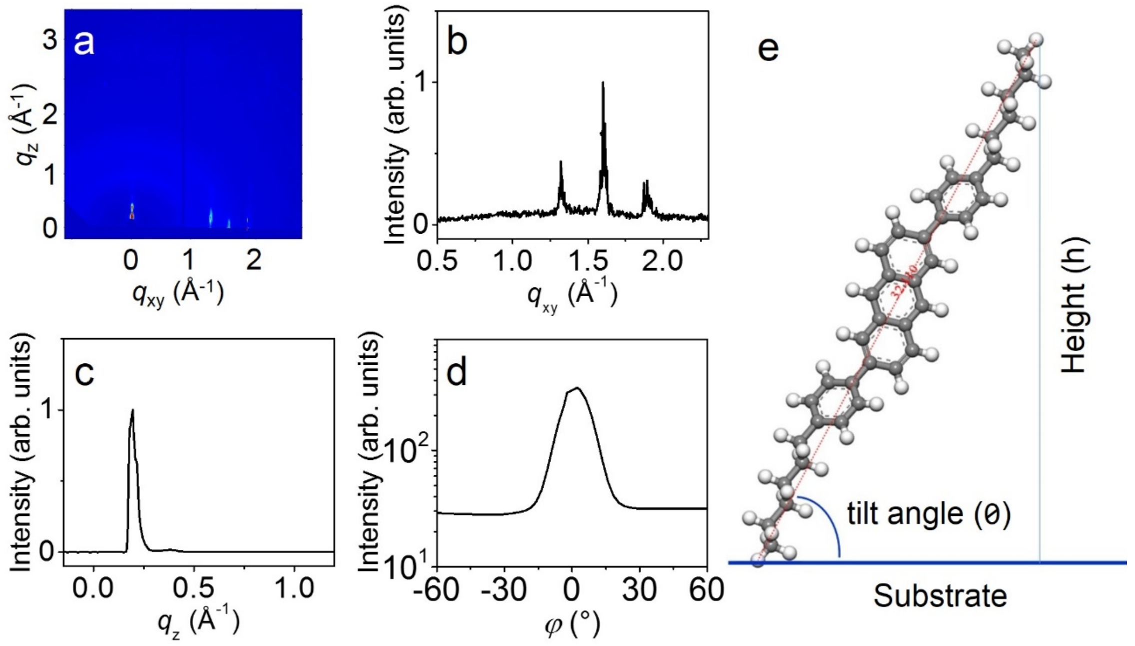

Supplementary Figure 8. The GIWAXS characterization and schematic of geometrical structures of MMCs. a, Two-dimensional GIWAXS pattern of C_6DPA MMCs. **b**, **c**, The corresponding profiles along (**b**) in-plane and (**c**) out-of-plane directions. **d,** Orientation distribution of C_6DPA molecules in MMCs for the out-of-plane (100) direction. **e**, The schematic of geometrical structures of C_6DPA on the substrates.

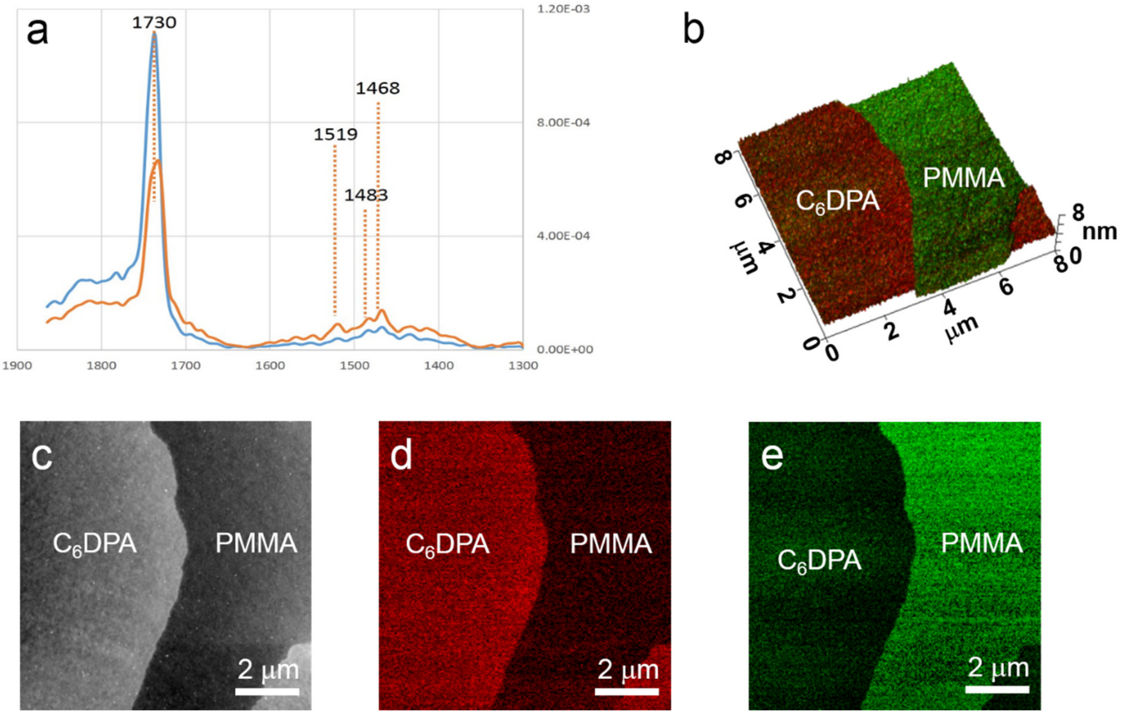

Supplementary Figure 9. Photo-induced force microscopy of MMCs. a, IR data of C_6DPA (orange) and PMMA (blue) where the absorption peaks at 1519 cm^{-1} and 1730 cm^{-1} are the characteristic peaks of C_6DPA and PMMA, respectively. **b**, 3D analysis of PiFM images at 1519 cm^{-1} and 1730 cm^{-1}, corresponding to the absorption peaks of C_6DPA (red) and PMMA (green), respectively. **c**, AFM image. **d,e**, PiFM image of C_6DPA and PMMA, respectively.

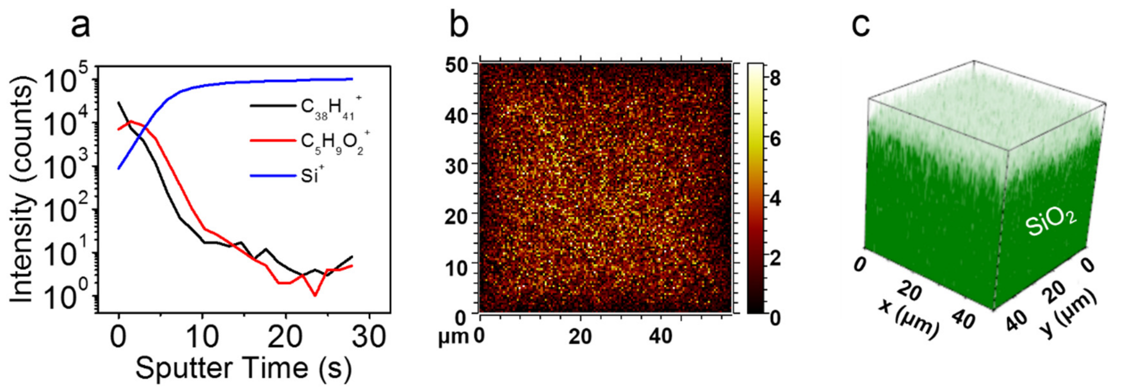

Supplementary Figure 10. TOF-SIMS characterization of MMCs. a, TOF-SIMS depth profiles of MMCs where the characteristic positive ions are $C_{38}H_{41}^+$ (C_6DPA), $C_5H_9O_2^+$ (PMMA), Si^+ (SiO_2). **b**, Two-dimensional TOF-SIMS images of $C_5H_9O_2^+$ (PMMA). **c**, 3D analysis of Si^+ (SiO_2).

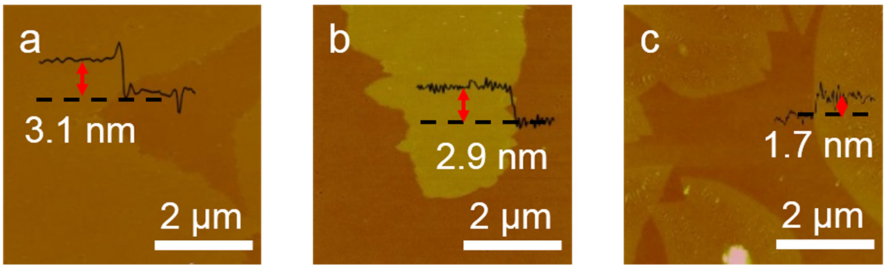

Supplementary **Figure 11. AFM images of various MMCs.** AFM images of MMCs: **a**, HTEB, **b**, C_8BTBT and **c**, NDI, respectively. See more details in Supplementary methods.

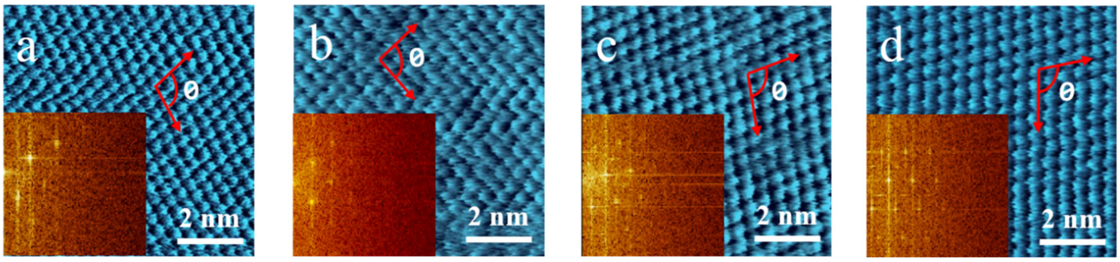

Supplementary Figure 12. HR-AFM images of various MMCs. HR-AFM images of MMCs: **a**, C_6DPA, **b**, NDI, **c,** HTEB and **d**, C_8BTBT, respectively. The insert pictures are corresponding 2D Fourier transfer patterns.

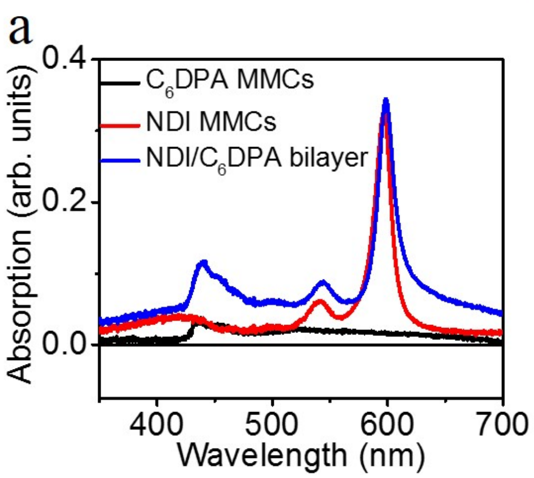

Supplementary Figure 13. The absorption and fluorescence spectrum. a, Absorption spectra of C_6DPA MMCs, NDI MMCs and p-n heterojunction. **b**, Fluorescence spectrum of NDI MMCs located on PMMA and C_6DPA respectively, at excitation wavelength of 530 nm.

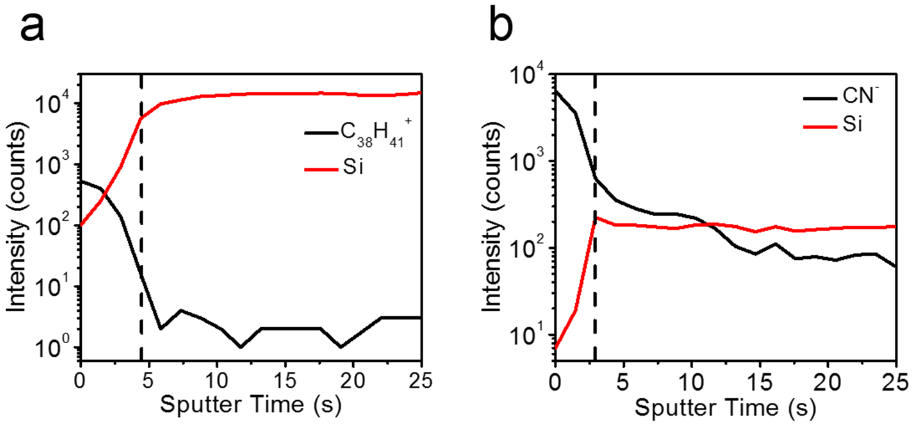

Supplementary Figure 14. The TOF-SIMS depth profiles of MMCs. The TOF-SIMS depth profiles of MMCs in (**a**) positive ions mode and (**b**) negative ions mode. The marked positive ions are $C_{38}H_{41}^{+}$ (C_6DPA), Si (SiO_2), CN^{-} (NDI). The TOF-SIMS characteristics were carried out on two adjacent heterojunctions because we cannot collect positive ions and negative ions at the same time. We find that the intensity of C_6DPA and NDI becomes weak at 4.43 s and 2.87 s, respectively. It means that the thickness of C_6DPA and NDI are 2.78 nm (4.43 s) and 1.8 nm (2.87 s), respectively, which are consistent with the values obtained from TEM data.

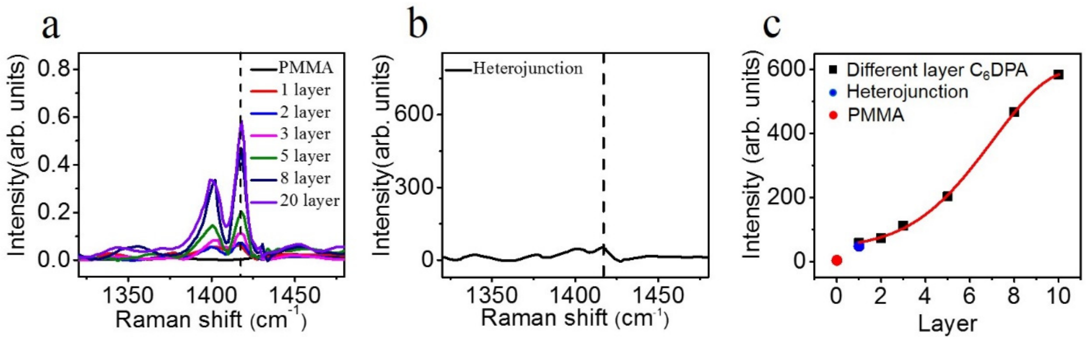

Supplementary Figure 15. The Raman spectrum of C_6DPA crystals. **a**, Raman spectrum of different layers of C_6DPA. The feature peak is at 1417 cm^{-1}. **b**, Raman spectra of C_6DPA in a p-n heterojunction. **c**, Raman intensity versus C_6DPA layer where red line is the fitted curve.

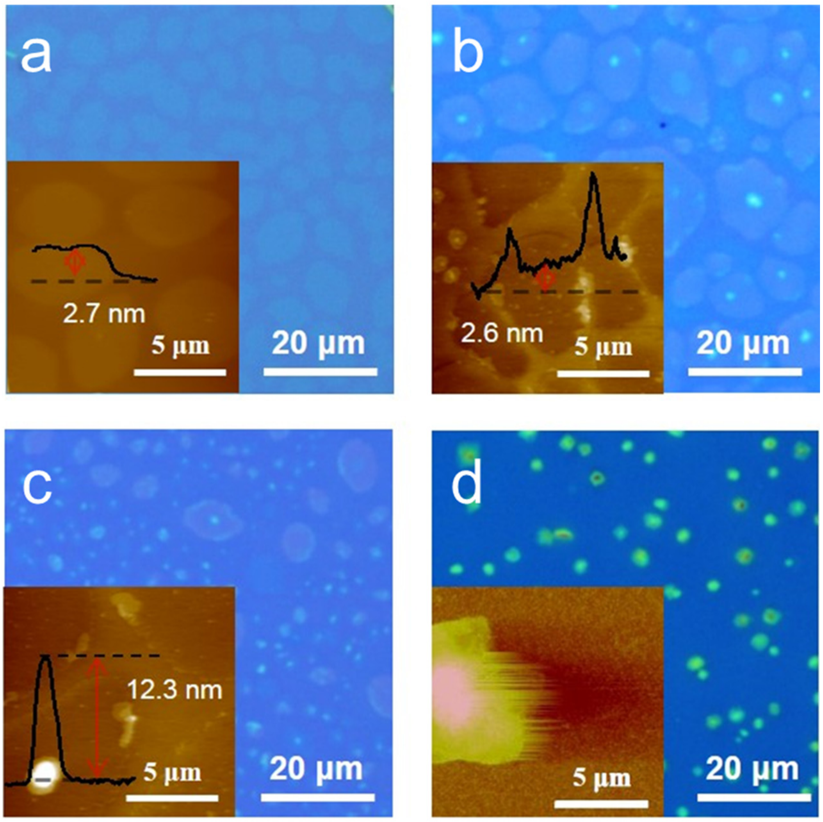

Supplementary Figure 16. Characterization of C_6DPA with different polymers. a-d, Optical images of C_6DPA. The corresponding polymers are P3HT (**a**, regiorandom), P3HT (**b**, regioregular), P3HT (**c**, regular) and PBTTT-C14 (**d**), respectively. The insert pictures are corresponding AFM images.

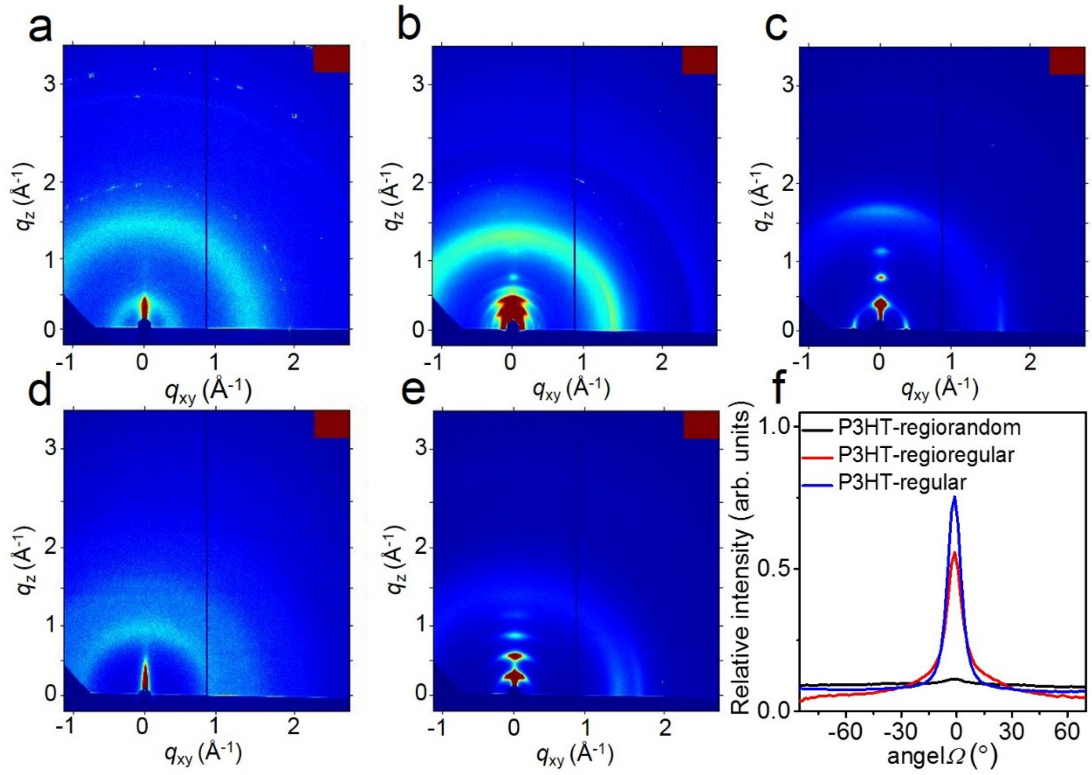

Supplementary **Figure 17. The GIWAXS characterization of different polymer films**. GIWAXS patterns of different polymer films: **a,** P3HT (regiorandom), **b,** P3HT (regioregular), **c**, P3HT (regular), **d,** PMMA, **e,** PBTTT-C14. **f**, The relative intensity of P3HT in the (200) packing versus angle.

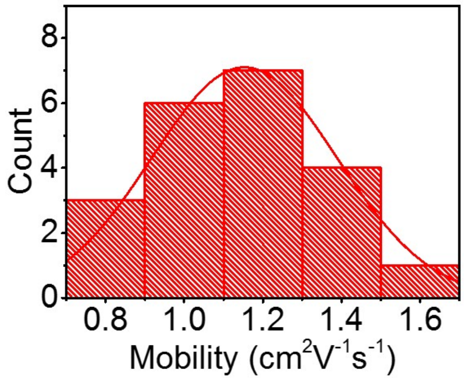

Supplementary Figure 18. The mobility statistics of the OFETs based on C_6DPA MMCs.

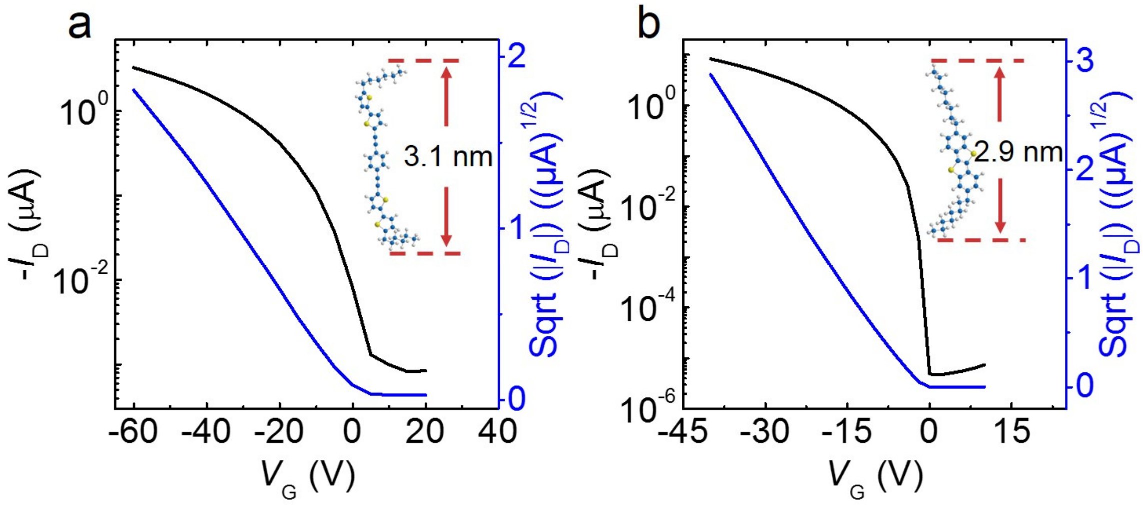

Supplementary **Figure 19. OFETs characteristics of other MMCs. a, b,** Transfer curves of HTEB and C_8BTBT MMCs devices.

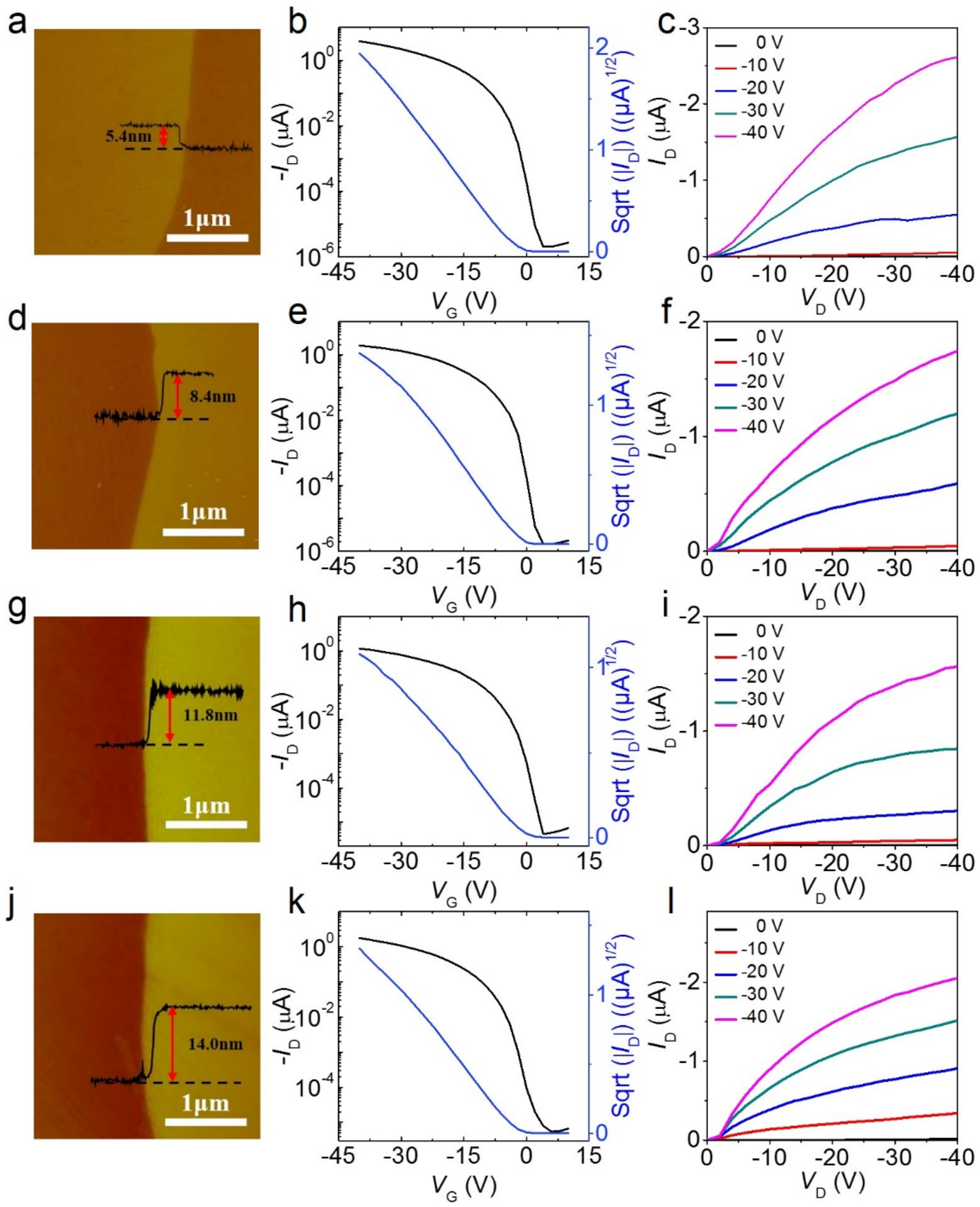

Supplementary Figure 20. OFETs characteristics of thick C_6DPA films on SiO_2/Si^{++} substrates. AFM images, transfer curves and output curves of (**a-c**) double-layer thick films, (**d-f**) three-layer thick films, (**g-i**) four-layer thick films and (**j-l**) five-layer thick films, respectively.

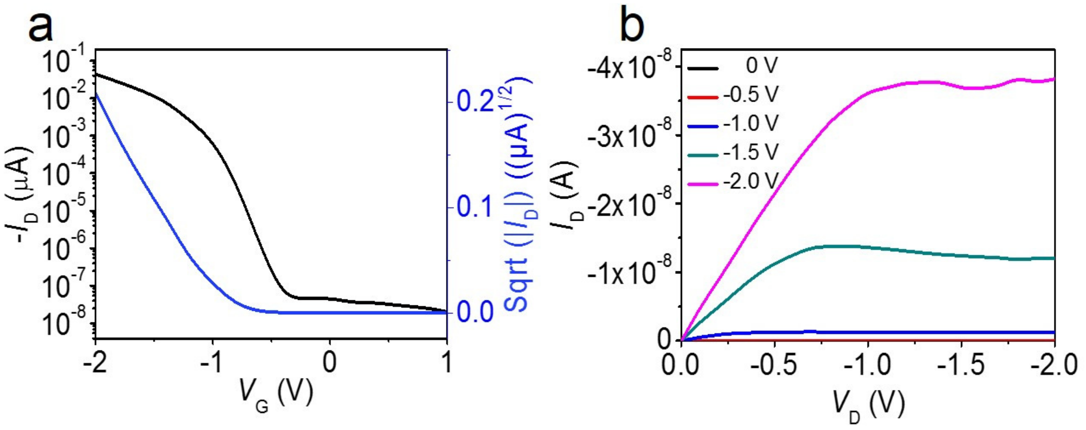

Supplementary Figure 21. Characteristics of ultralow operation voltage device. a, Transfer curve and **b,** output curve FETs characteristics of devices on SiO_2/Si^{++} substrates.

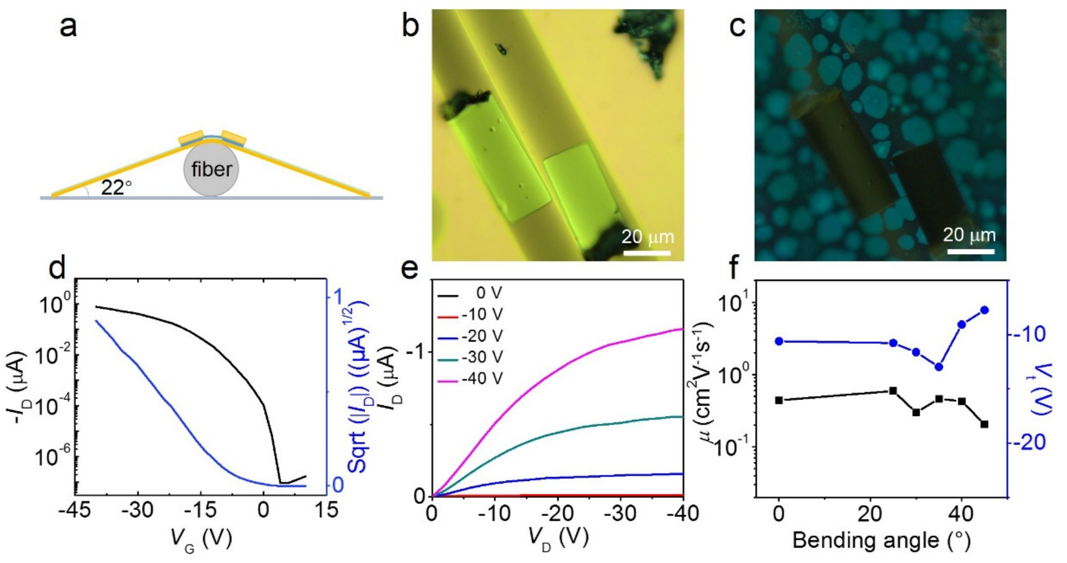

Supplementary Figure 22. The characterization of flexible device. a, Schematic diagram of the flexible device (the gray color, yellow color and blue color represent fiber, gold and C$_6$DPA, respectively). **b**,**c**, The optical image and fluorescent image of bending C$_6$DPA with a bending angle of ~44°. **d**,**e**, The corresponding transfer and output curves. **f**, The mobility with different bending angles.

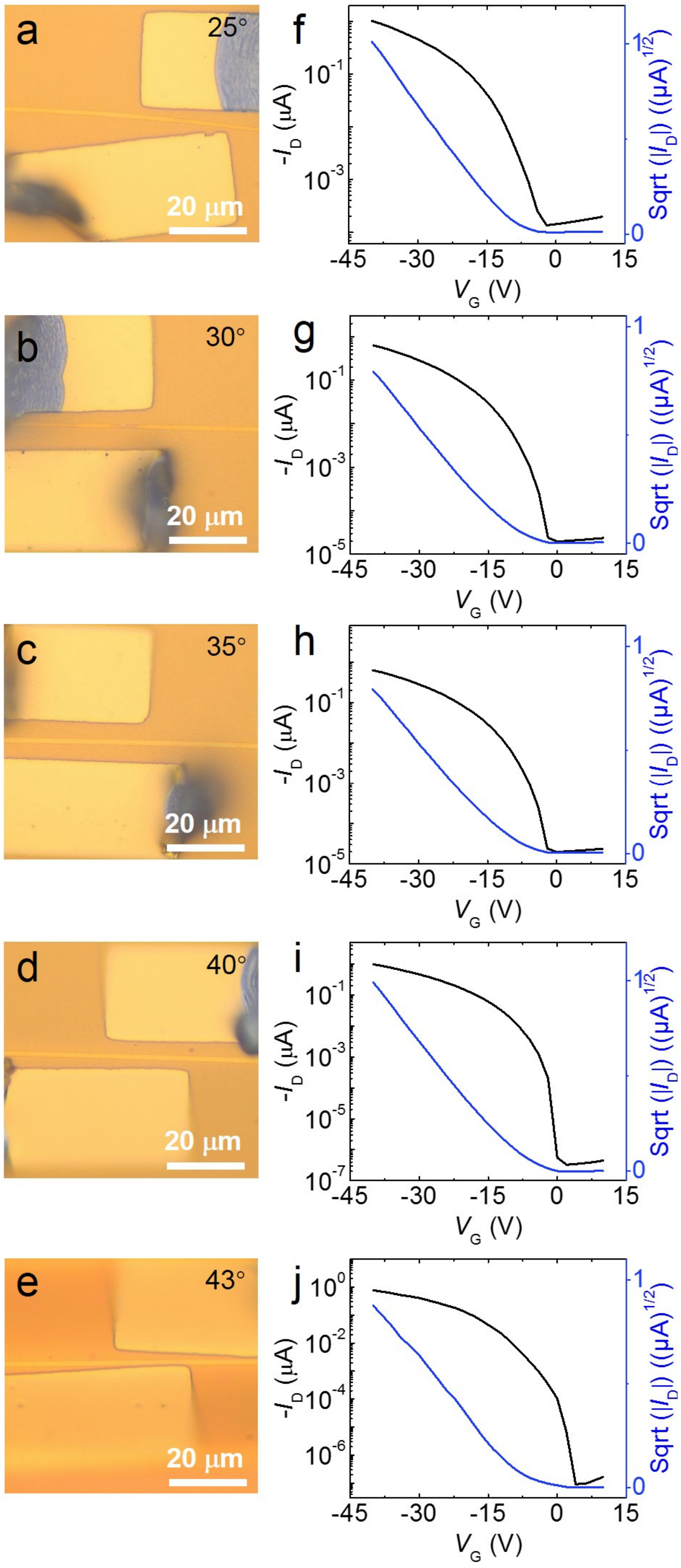

Supplementary Figure 23. The OFETs characteristics of flexible device. a-e, The optical images of MMCs with different bending angles. **f-j**, Corresponding transfer curves.

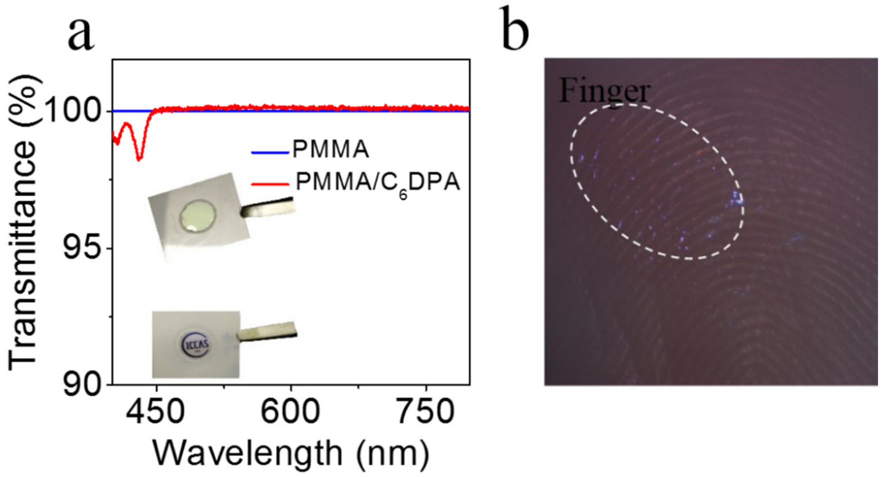

Supplementary Figure 24. The characterization of transparent device. a, The transmittance of PMMA/C_6DPA. The inserted pictures are demonstration for PMMA/C_6DPA. **b**, Optical images of the films transferred on the skin of finger.

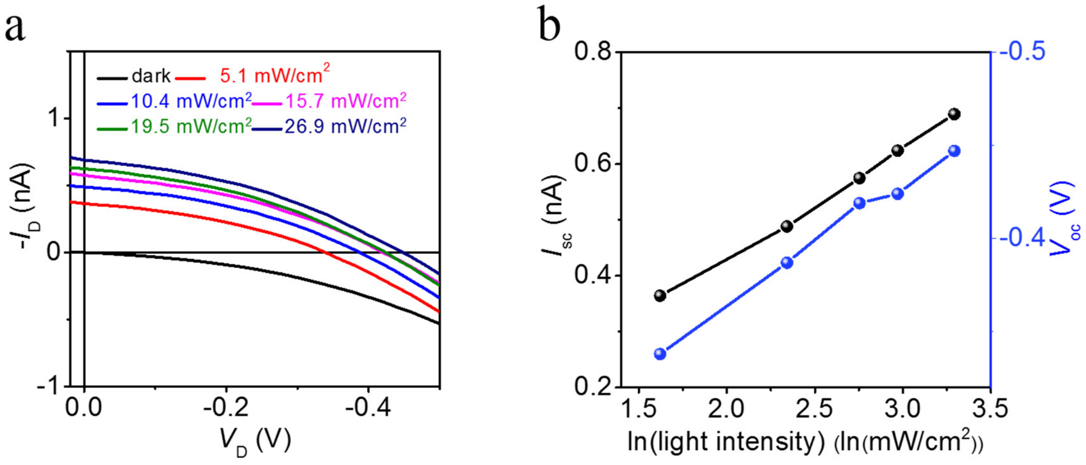

Supplementary Figure 25. **Current-voltage characteristics of the device. a**, Current-voltage characteristics of the device under different light intensity at V_G= -80 V. **b**, The extracted short circuit currents (I_{sc}) and open circuit voltages (V_{oc}).

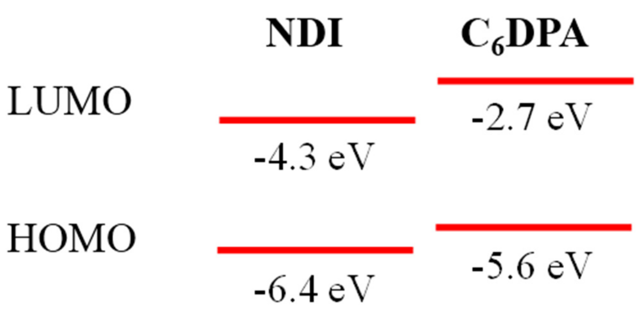

Supplementary Figure 26. The schematic diagram of energy level distribution of NDI and C_6DPA.

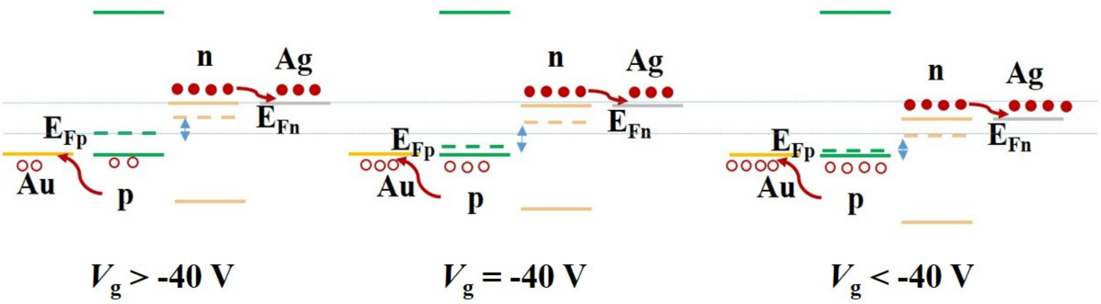

Supplementary **Figure 27**. **The band diagrams of the OPV under different V_g.** Note that here we neglect the effects of gate electric field on the density of electrons in the NDI semiconductor, and on the band bending at the metal/semiconductor contacts, because these effects are not the dominant ones in such cases. The exact and detailed mechanisms require further investigation.

Supplementary Tables

Lattice	C_6DPA	NDI	HTEB	C_8BTBT
b	0.45 nm	0.45 nm	0.77 nm	0.80 nm
c	0.47 nm	0.46 nm	0.60 nm	0.60 nm
θ	99.5°	99.1°	90.5°	90.3°

Supplementary Table 1. The lattice parameters of C_6DPA MMC, NDI MMC, HTEB MMC and C_8BTBT MMC.

Supplementary Notes

Supplementary Note 1: Optimization of MMCs.

In order to obtain high quality and large size MMCs, we explored the variation of the size and crystallinity of MMCs with different parameters such as the concentration of PMMA and C_6DPA (Supplementary Figure 1-6). From the studies of variation of PMMA concentration (Supplementary Figure 1-4), it can be concluded that, a low PMMA concentration of 4 mgmL^{-1} could promise the formation of C_6DPA crystals with confined thickness of a monolayer. As shown in Supplementary Figure 1f, increase in PMMA concentration is accompanied with a first increase and then decrease in C_6DPA crystal size, featuring a peak average grain size of ~40 μm at 4 mgmL^{-1}. This phenomenon can be interpreted as follows: at very low PMMA concentration (< 4 mgmL^{-1}), the C_6DPA does not form continuous films due to the dewetting effect of the substrates. At a moderate PMMA concentration (i.e. about 4 mgmL^{-1}), polymer-assisted wetting of the film could induce the gradual formation of MMCs through vertical phase separation, due to its higher surface energy of PMMA as compared to that of C_6DPA. With PMMA concentration further increased (>4 mgmL^{-1}), PMMA solidified and the polymer chain entanglement may restrain the diffusion and crystal growth of C_6DPA molecules, leading to rather small crystallites. Furthermore, we also investigated the effect of C_6DPA content on forming MMCs by maintaining PMMA concentration at 4 mgmL^{-1}, as shown in Supplementary Figure 1g and Supplementary Figure 5-6. The crystal size experiences an increase with the increase of C_6DPA concentration, following with a declining afterward. This is because at the moderate concentration (i.e. 20% C_6DPA content), the solution remains at a "meta-stable" state for a sufficiently long time before entering the "supersaturated" state, where spontaneous nucleation occurs, resulting in formation of large crystals. The maximum size and the high quality of crystals (Supplementary Figure 1) demonstrated

that the optimized concentration of PMMA is 4 $mgmL^{-1}$ at C_6DPA weight concentration of 20 wt.%.

Supplementary Note 2: Tilted angle of the C_6DPA molecules in MMC.

The geometrical structure of C_6DPA optimized by DFT at the B3LYP/6-311G(d) level in Guassian16 Program shows molecular length of about 3.21 nm. In addition, from the GIWAXS pattern, a diffraction peak in the out-of-plane direction is observed, and the measured spacing along the *a* axis is 3.24 nm. Hence, the GIWAXS and calculated results together indicate that the C_6DPA molecules in thick crystals are perpendicular to the substrates. However, the thickness of the molecular layer in the MMCs is only 2.7±0.1 nm (Figure 1c) according to AFM measurements, smaller than molecular length of C_6DPA (3.2 nm) obtained from theoretical and GIWAXS results, indicating a tilted angle of 57.6°. Here the tilt angle θ is calculated by the following equation: $h/l=\sin\theta$, where h is the measured height of the monolayer and l is the calculated molecular length of C_6DPA (Supplementary Figure 8e).

Supplementary Note 3: Photo-induced force microscopy (PiFM) and time of flight secondary ion mass spectrometry (TOF-SIMS) results.

According to the AFM results, it seems that the C_6DPA MMCs assembled on the PMMA surface. However, it is not clear whether the MMCs are only distributed on the surface of PMMA or also embedded within the PMMA domain. To answer this question, photo-induced force microscopy (PiFM) measurement was carried out. The absorption peaks at 1519 cm^{-1} and 1730 cm^{-1} extracted from the infrared spectroscopy (Supplementary Figure 9a) are signature peaks of C_6DPA and PMMA, respectively. As shown in Supplementary Figure 9b-9e, the distinct boundary between the MMCs and PMMA domains is clearly observed, which implies that there is no C_6DPA residue in PMMA. However, the AFM images and PiFM images can only reveal the planar distribution of MMCs. In order to reveal the vertical distribution of C_6DPA, time of flight secondary ion mass spectrometry (TOF-SIMS) full-depth profiles were recorded in the positive ion mode (Figure 2a-2d). As shown in Supplementary Figure 10, positive ions $C_{38}H_{41}^+$ and $C_5H_9O_2^+$ are the characteristic ions of C_6DPA and PMMA, respectively. Through a 3D analysis of the $C_{38}H_{41}^+$, $C_5H_9O_2^+$, Si^+ and total ions (Figure 2b-2d and Supplementary Figure 10c), it can be clearly seen that C_6DPA MMCs distribute only on the surface of PMMA, where the red color, blue color and green color represent C_6DPA, PMMA and Si, respectively. These results demonstrate that C_6DPA MMCs are exclusively distributed on the top of PMMA layer rather than on the surface of SiO_2/Si^{++} substrates or inside the PMMA.

Supplementary Note 4: The characterization of the p-n heterojunction based on C_6DPA and NDI MMCs.

According to the AFM images, the thickness of the upper NDI crystal is only about 1.7 nm, shorter than the molecular length of NDI, indicating that the NDI layer is indeed a monolayer crystal. Absorption spectra on the NDI indicates the zone is a heterojunction including both C_6DPA and NDI molecules (Supplementary Figure 13), which indicates that there should be a C_6DPA layer located underneath the NDI one. In order to determine the thickness of C_6DPA, TEM and TOF-SIMS characterizations were performed (shown in Figure 1l and Supplementary Figure 14). The sectional image of the heterojunction shows the thickness of C_6DPA is about 2.8 nm, which is thickness of a monolayer. The thickness of the NDI layer is found to be about 1.8 nm, which is consistent with the one obtained from the AFM results. Moreover, Raman intensity is dependent on the thickness of C_6DPA crystals, which provide an additional way to detect the thickness of bottom layer C_6DPA crystals. The embedded bottom C_6DPA MMC has been verified by thickness effect on intensity of Raman spectra as shown in Supplementary Figure 15. The HR-AFM results shown in Figure 1m-1n demonstrate the crystallinity of the C_6DPA and NDI layers. These results indicate that p-n heterojunctions consisting of p- and n-type MMCs have been successfully prepared.

Supplementary Note 5: Different layers growth by Solvent Vapor Annealing method.

We also prepared C_6DPA crystals with different layers through the method of Solvent Vapor Annealing (SVA), as shown in Supplementary Figure 20. The prepared MMCs were annealed in saturated chlorobenzene atmosphere at room temperature for 3 hours, after which multilayers of C_6DPA could be obtained. The exact thickness of layers of the C_6DPA crystals were measured by AFM, and corresponding number of layers was estimated. The electrical performance of C_6DPA crystals with different layers are shown in Supplementary Figure 20. To our surprise, the MMCs have higher mobility compared with the thick crystals prepared by SVA (Figure 3c). The results indicate the fact that charge transport in OFET occurs at the interface between gate dielectrics and the organic semiconductor within the thickness of a monolayer.

Supplementary Note 6: Characterization of transparent, flexible devices.

The ultra-thin nature of MMCs makes them possible for fabrication of ultra-thin, transparent, flexible and wearable devices. The traditional method for fabricating flexible devices is depositing organic polymer materials on flexible substrates and the flexibility is demonstrated by bending or twisting the devices. In order to characterize the high flexibility of MMCs, we transferred the MMCs onto SiO_2/Si^{++} substrates coated with ultra-fine carbon fibers. As shown in Supplementary Figure 22, the fluorescent image and optical image demonstrate the C_6DPA MMCs immobilized robustly onto the carbon fiber with small radius of around 8 μm, which implies their good flexibility. According to Supplementary Figure 23, the calculated maximum bending angle could reach 44°. Although the radius of the curvature cannot be exactly determined, the results still imply the excellent bendability of MMC crystals (especially for small size crystals). The bended MMC device shows a mobility of 0.21 $cm^2V^{-1}s^{-1}$ (bending angle = 44°), which is comparable to the mobility value of the device prior to transferring (0.23 $cm^2V^{-1}s^{-1}$). In addition, the MMCs exhibit excellent transmittance (almost 100% with the minimum 98% in the range of 400-450 nm) (Supplementary Figure 24). The results demonstrate that the method could be employed to fabricate high-performance, ultra-flexible and transparent MMCs FETs. Furthermore, the MMCs show good fit with the skin, which is particularly important for wearable devices (Supplementary Figure 24).

Supplementary Methods

The phase separation occurs at the ultra-thin two-dimensional space, and thus named as two-dimensional space phase separation method (see the schematic diagram in Supplementary Figure 1). In this study, to improve the surface wetting, all the substrates have been treated by oxygen plasma (Plasma Etch INC. PE-25) at 100 W for 5 min. Then, a solution mixture of poly(methyl methacrylate) (PMMA, Mw=350000, Sigma Aldrich) and 2,6-bis(4-hexylphenyl)anthracene (C_6DPA) in chlorobenzene was spin-coated on SiO_2/Si^{++} substrates in ambient conditions, after which well-defined films were obtained directly at the marginal zone of the substrates (Figure 1 and Supplementary Figure 1-7). It can be concluded that monolayer films could be prepared when the thickness of the blending films is down to about 10 nm (Supplementary Figure 2-3). Polymer materials of poly(3-hexylthiophene) (P3HT) (regiorandom, Mw 20000~45000, Sigma Aldrich), P3HT (regioregular, Mw 20000~45000 with regularity≥90%, Sigma Aldrich), P3HT (regular, Mw 36000~58000 with regularity≥95%, Rieke Metals Inc) and poly[2,5-bis(3-tetradecylthiophen-2-yl)thieno[3,2-b]thiophene] (PBTTT-C14) (Mw>16000, Betterchem) are commercially available.

In order to get detail information of monolayer molecule crystals (MMCs), the time of flight secondary ion mass spectrometry (TOF-SIMS), photo-induced force microscopy (PiFM), grazing incidence wide angle X-ray scattering (GIWAXS) and high-resolution atomic force microscopy (HR-AFM) were conducted. The lattice constants of the films along the *b* and *c* axes are 0.45 nm and 0.47 nm, respectively, with a θ~99.5° (HR-AFM image, Figure 1d). As shown in the 2D GIWAXS profiles (Supplementary Figure 8) along in-plane and out-of-plane directions, the lamella thickness and the π-stacking distance are estimated to be 3.24 nm and 0.47 nm, respectively, which are consistent with the parameters obtained from the HR-AFM measurement (Figure 1d). The coherence length is about 13 nm, demonstrating a highly ordered structure within the molecular layer. However, due to the co-existence of a few number of multi-layer structures, a diffraction peak is observed in out-of-plane directions in GIWAXs. All these results together confirm the monolayer molecular crystal structures.

Molecular dynamics (MD) simulations were performed by the Gromacs-4.6.7 software package with the general AMBER force field[1,2]. Firstly, we obtained a PMMA amorphous film by using a high-temperature annealing method[3] , which includes 100 PMMA chains (each has 20 repeat units) and has a final box size of $8 \times 8 \times 5.9$ nm^3. Then, we constructed

a C_6DPA/CB solution containing 200 C_6DPA molecules and 5000 CB molecules, which was equilibrated at 300 K and 1 bar for 10 ns (with the final box size: 8 × 8 × 21.8 nm^3). Subsequently, the C_6DPA/CB solution was placed on top of the PMMA with the z-direction elongated to 35 nm to generate a large empty space. The solvent evaporation process was mimicked by a quasi-equilibrium approach[4] through removing the gas-phase solvent molecules every 100 ps (until no new gas-phase solvent molecules appeared). To accelerate this process, the temperature was increased to 400 K. After 35 ns of solvent evaporation, 5 ns of equilibration was carried out at 300 K. The tilt angle of the equilibrated MMC was calculated by averaging the tilt angles of the MMC molecules. The geometrical structures of C_6DPA further optimized at B3LYP/6-311G(d) level in Guassian16 Program.

Supplementary References

1. Hess, B.; Kutzner, C.; van der Spoel, D.; Lindahl, E. GROMACS 4: Algorithms for Highly Efficient, Load-Balanced, and Scalable Molecular Simulation. *J. Chem. Therory Comput.* **4**, 435-447 (2008).
2. Wang, J.; Wolf, R. M.; Caldwell, J. W.; Kollman, P. A.; Case, D. A. Development and Testing of a General Amber Force Field. *J. Comput. Chem.* **25**, 1157-1174 (2004).
3. Han, G.; Guo, Y.; Song, X.; Wang, Y.; Yi, Y. Terminal π-π Stacking Determines Three-Dimensional Molecular Packing and Isotropic Charge Transport in an A-π-A Electron Acceptor for Non-Fullerene Organic Solar Cells. *J. Mater. Chem. C* **5**, 4852-4857 (2017).
4. Han, G.; Shen, X.; Duan, R.; Geng, H.; Yi, Y. Revealing the Influence of the Solvent Evaporation Rate and Thermal Annealing on the Molecular Packing and Charge Transport of DPP(TBFu)2. *J. Mater. Chem. C* **4**, 4654-4661 (2016).
